# Fixed/Predefined-time synchronization of memristor-based complex-valued BAM neural networks for image protection

**DOI:** 10.3389/fnbot.2022.1000426

**Published:** 2022-10-17

**Authors:** Aidi Liu, Hui Zhao, Qingjie Wang, Sijie Niu, Xizhan Gao, Zhen Su, Lixiang Li

**Affiliations:** ^1^Shandong Provincial Key Laboratory of Network Based Intelligent Computing, School of Information Science and Engineering, University of Jinan, Jinan, China; ^2^College of Computer Science and Technology, Chongqing University of Posts and Telecommunications, Chongqing, China; ^3^State Key Laboratory of Networking and Switching Technology, Information Security Center, Beijing University of Posts and Telecommunications, Beijing, China

**Keywords:** fixed-time synchronization, predefined-time synchronization, bidirectional associative memory, image encryption and decryption, complex-valued neural networks

## Abstract

This paper investigates the fixed-time synchronization and the predefined-time synchronization of memristive complex-valued bidirectional associative memory neural networks (MCVBAMNNs) with leakage time-varying delay. First, the proposed neural networks are regarded as two dynamic real-valued systems. By designing a suitable feedback controller, combined with the Lyapunov method and inequality technology, a more accurate upper bound of stability time estimation is given. Then, a predefined-time stability theorem is proposed, which can easily establish a direct relationship between tuning gain and system stability time. Any predefined time can be set as controller parameters to ensure that the synchronization error converges within the predefined time. Finally, the developed chaotic MCVBAMNNs and predefined-time synchronization technology are applied to image encryption and decryption. The correctness of the theory and the security of the cryptographic system are verified by numerical simulation.

## 1. Introduction

After entering the twenty-first century, brain-like intelligence and neural network have developed rapidly. With the support of technologies such as artificial intelligence, deep learning, and cloud computing, corresponding achievements have emerged in an endless stream (Su et al., 2021; Wen and Su, 2022), which also poses greater challenges to the large-scale information processing capacity of computing systems. Many researchers have explored the direction of brain-like research (Rubinov and Sporns, 2010; Zhao H. et al., 2021), trying to get inspiration from the structure of the human brain and the way of information processing. Associative memory is one of the most active behaviors in the human brain, and it simulates the ability of the real nervous system to process information. In 1988, Kosko extended the traditional Hopfield neural networks and established bidirectional associative memory (BAM) neural networks (Kosko, 1988). The networks have a relatively complex structure, and their neurons are distributed in two layers. Each neuron in each layer is connected to all neurons in the other layer, while all neurons in the same layer are not connected to each other. At present, with the rapid development of artificial intelligence, the synchronous control and stability analysis of BAM neural networks has become the mainstream research direction. Researchers have invested a lot of time and energy to explore BAM neural networks, which provides a new research idea for the theoretical analysis of complex networks (Ke and Miao, 2013; Zhang et al., 2014; Qi et al., 2015; Zhang and Quan, 2015; Zhang and Yang, 2020; Zhao Y. et al., 2021; Liu et al., 2022).

In recent years, the development of many industrial products has involved complex signal problems. The introduction of complex signals extends the state variables of the controlled system from the real domain to the complex domain, which leads to an upsurge of research on complex-valued neural networks. The state variables, connection weights, and activation functions of complex-valued neural networks are complex numbers, which can solve problems that cannot be solved by real-valued neural networks, such as XOR and symmetry detection. The complex-valued neural networks have more advantages in network learning ability and self-organization. At present, some interesting results have been proposed (Liu et al., 2017; Zhang et al., 2018; Li and Mu, 2019; You et al., 2020).

The memristor was originally predicted by Chua (1971). HP laboratory first developed the memristor components of nanometer size in 2008 (Strukov et al., 2008). The resistance of the memristor will vary with the charge flow and can remain unchanged after power failure. In addition, the memristor is considered to be a perfect device for simulating synapses due to its advantages of nanometer size, low power consumption, and easy large-scale integration. There has been much interesting research on the dynamics of memristive neural networks (Li and Cao, 2016; Wang et al., 2017; Yang et al., 2020). Compared with traditional artificial neural networks, memristor neural networks can better simulate the structure and function of the human brain.

As one of the dynamic behaviors, synchronization behavior describes the cooperative consistency in a group, which is manifested in the network as the cooperative and consistent relationship after the interaction of node states. Fixed-time synchronization is a special kind of finite-time synchronization. The corresponding synchronization time has a certain upper bound, which is not dependent on the initial value of the system but only related to the system parameters and the controller. At present, there are some research results on fixed-time synchronization. Cao and Li (2017) studied a fixed-time synchronization control method based on memristor and recurrent neural networks with time delay and estimated the settling time of fixed-time synchronization. Chen et al. (2019) derived a new fixed-time stability theorem, and sufficient conditions were derived to guarantee the fixed-time synchronization of neural networks. Yang et al. (2019) investigated the fixed-time synchronization of memristor-based neural networks with time-delay and coupling. The research of fixed-time stability promotes the development of many practical applications. In some practical engineering applications, the system is required to reach the origin in the specified time, but the main disadvantage of fixed-time stability is that the relationship between the system parameters and the convergence time is not clear. Then, Sanchez-Torres et al. (2014) proposed the definition of predefined-time stability, which could be solved by adjusting the parameters in the process of controller design. Therefore, the system can achieve stability before the predefined-time *Tc*. Predefined-time stability is the result of fixed-time stability optimization. At present, there are some research results on predefined-time synchronization. Lin et al. (2020) proposed a predefined-time stability theorem based on a piecewise Lyapunov function, in which the Lyapunov function should satisfy the inequality: $\overset{∙}{V}\left(t\right)\leq -\frac{G_{c}}{T_{c}}\left(\alpha V^{p}\left(t\right)+c\right)$ with α, *c, p* > 0, *G*_*c*_ is the minimum upper bound for fixed-time stability and *T*_*c*_ is a custom parameter. Aldana-Lopez et al. (2019) studied more relaxed predefined time stability conditions, where the Lyapunov function should satisfy the inequality $\overset{∙}{V}\left(t\right)\leq -\frac{G_{c}}{T_{c}}\left(\alpha V^{p}\left(t\right)+\beta V^{q}\left(t\right)\right)$ with α, β > 0, *p*> 1 and 0 < *q* < 1. Anguiano-Gijon et al. (2019) introduced *G*_*c*_ into inequality in the form of $\overset{∙}{V}\left(t\right)\leq -\frac{\pi }{qT_{c}}\left(V^{1-\frac{q}{2}}\left(t\right)+V^{1+\frac{q}{2}}\right)$ with 0 < *q* < 1. The predefined-time stability theorem proposed in this paper is more general than (Aldana-Lopez et al., 2019; Anguiano-Gijon et al., 2019; Lin et al., 2020). Synchronization has important applications in many fields, such as secure communication, nonlinear control systems, pattern recognition, and information processing (Alimi et al., 2019; Ouyang et al., 2020). Synchronization also plays an important role in laser systems, superconducting materials, and conventional bus dispatching (Gkiotsalitis et al., 2020; Wang et al., 2021). In addition, Su et al. (2020) studied the manipulator control based on an improved recurrent neural network. The ultimate end-effector tracking error can reach asymptotic convergence, which is also a concrete manifestation of synchronous control.

This paper also studies the image encryption scheme based on MCVBAMNNs. As we all know, the research results of brain-like neural networks have shown great power in practical applications. Memristor-based neural networks, which are more similar to the structure of human synapses, also show unique functions and values in the application. The digital image is an important way to represent information, research on image data privacy protection based on a general memristor-based neural network learning mode has broad application prospects. However, there are still few studies on image data privacy protection using memristor-based artificial neural networks and their network behavior characteristics. As an ideal tool to simulate human neural networks, memristor-based neural networks can be used to maximize the ability of the human brain to recognize and classify, which shows the potential of memristor-based neural networks in pattern recognition. Recognition and classification of sensitive areas of digital images and proposing appropriate privacy protection solutions can give full play to the advantages of memory-based neural networks in recognition and classification and have a wide range of applications.

Motivated by the above discussions, we investigated the fixed-time synchronization and the predefined-time synchronization of MCVBAMNNs with leakage time-varying delay. The innovations of this paper are presented as follows: First, based on the appropriate fixed-time stability lemma, the feedback controller is designed, and the fixed-time synchronization problem of MCVBAMNNs is studied. By comparison, the results of this paper are less conservative. Second, a new predefined-time stability theorem is introduced, where the predefined time is set more flexibly and in a more general form. Thirdly, a more simple and effective discontinuous controller is designed, and sufficient conditions for MCVBAMNNs to achieve predefined-time synchronization are obtained. The synchronization time does not depend on the initial value and can be adjusted according to the controller parameters. Finally, an image encryption and decryption scheme based on predefined-time synchronization is presented, and the predefined time can be used as the secret key. Numerical simulation verifies the validity of the theoretical results and the feasibility of the encryption scheme.

*Notations:* In this study, ℝ, ℂ, ℝ^*n*^, and ℂ^*n*^ represent the real field, complex field, *n*-dimensional real space, and *n*-dimensional complex space, respectively. *u* = *R* + *I**i*** ∈ ℂ, where ***i*** meets $\text{i}=\sqrt{-1}$.

## 2. Problem formulation and preliminaries

We consider the following MCVBAMNNs as the drive system, which is given as:


(1) $$\begin{array}{l} \{\begin{array}{c} {\dot{x}}_{1i}^{u}\left(t\right)=-\eta_{i}^{u}\left(x_{1i}^{u}\left(t-\tau \left(t\right)\right)\right)x_{1i}^{u}\left(t-\tau \left(t\right)\right)+\overset{m}{\underset{j=1}{\sum }}a_{ji}^{u}\left(x_{1i}^{u}\left(t\right)\right)f_{j}^{u}\left(x_{2j}^{u}\left(t\right)\right)\\ \;+\overset{m}{\underset{j=1}{\sum }}b_{ji}^{u}\left(x_{1i}^{u}\left(t-\tau \left(t\right)\right)\right)f_{j}^{u}\left(x_{2j}^{u}\left(t-\sigma \left(t\right)\right)\right),\\ {\dot{x}}_{2j}^{u}\left(t\right)=-\xi_{j}^{u}\left(x_{2j}^{u}\left(t-\sigma \left(t\right)\right)\right)x_{2j}^{u}\left(t-\sigma \left(t\right)\right)+\overset{n}{\underset{i=1}{\sum }}c_{ij}^{u}\left(x_{2j}^{u}\left(t\right)\right)g_{i}^{u}\left(x_{1i}^{u}\left(t\right)\right)\\ \;+\overset{n}{\underset{i=1}{\sum }}d_{ij}^{u}\left(x_{2j}^{u}\left(t-\sigma \left(t\right)\right)\right)g_{i}^{u}\left(x_{1i}^{u}\left(t-\tau \left(t\right)\right)\right), \end{array} \end{array}$$

where *i* = 1, 2, ..., *n*, *j* = 1, 2, ...*m*; $x_{1i}^{u}\left(t\right),x_{2j}^{u}\left(t\right)\in \mathbb{C}$ represent the voltage of the capacitor *i*th and *j*th nodes at time *t*. The initial values of the system (1) are $x_{1}^{u}\left(0\right)=\varphi_{1}^{u}\left(s\right)$ and $x_{2}^{u}\left(0\right)=\varphi_{2}^{u}\left(s\right)$, *s* ∈ ℝ; $f_{j}^{u}\left(\cdot \right)$ and $g_{i}^{u}\left(\cdot \right)$: ℂ → ℂ are complex-valued activation functions; τ(*t*) and σ(*t*) are the leakage time-varying delays satisfying 0 < τ(*t*) < σ(*t*) < *C* (*C* is a constant); $\eta_{i}^{u}>0$ and $\xi_{j}^{u}>0$ are the rates of neuron self-inhibition; $a_{ji}^{u}$, $b_{ji}^{u}$, $c_{ij}^{u}$, $d_{ij}^{u}$ are the memristive connection weights.

The parameters signification and performance of MCVBAMNNs are described as:


$$\eta_{i}^{u}\left(x_{1i}^{u}\left(t-\tau \left(t\right)\right)\right)=\frac{1}{C_{1i}}\left[\overset{m}{\underset{j=1}{\sum }}\left(M_{aji}+M_{bji}\right)sign_{ji}+\frac{1}{R_{1i}}\right],$$

$$\xi_{j}^{u}\left(x_{2j}^{u}\left(t-\sigma \left(t\right)\right)\right)=\frac{1}{C_{2j}}\left[\overset{n}{\underset{i=1}{\sum }}\left(M_{cij}+M_{dij}\right)sign_{ij}+\frac{1}{R_{2j}}\right],$$

$$a_{ji}^{u}\left(x_{1i}^{u}\left(t\right)\right)=\frac{sign_{ji}}{C_{1i}M_{aji}},\;b_{ji}^{u}\left(x_{1i}^{u}\left(t-\tau \left(t\right)\right)\right)=\frac{sign_{ji}}{C_{1i}M_{bji}},$$

$$c_{ij}^{u}\left(x_{2j}^{u}\left(t\right)\right)=\frac{sign_{ij}}{C_{2j}M_{cij}},\;d_{ij}^{u}\left(x_{2j}^{u}\left(t-\sigma \left(t\right)\right)\right)=\frac{sign_{ij}}{C_{2j}M_{dij}}.$$

**Figure 1:**
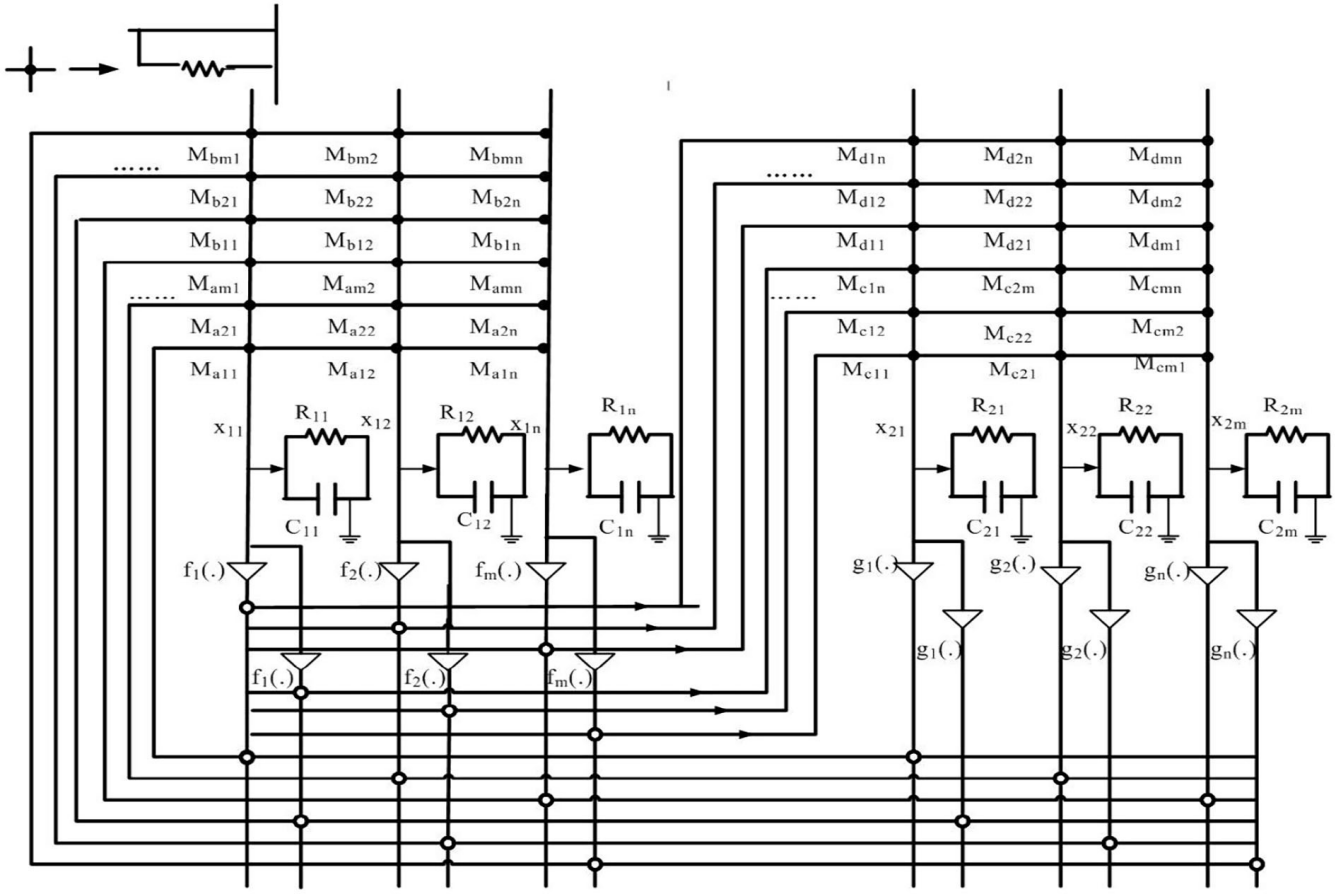
Circuit of memristor-based BAMNNs.

The memristor-based BAM neural networks model can be implemented by very large-scale integration (VLSI) circuits as shown in Figure 1. Taking a real-valued system as an example, where *sign*_*ij*_ = *sign*_*ij*_ = 1 if *i* ≠ *j*, otherwise *sign*_*ij*_ = *sign*_*ij*_ = −1, *x*_1*i*_(·) and *x*_2*j*_(·) represent the state of the subsystems, *f*_*j*_(·) and *g*_*i*_(·) are amplifiers, *M*_*aji*_ is the connection memristor between the amplifier *f*_*j*_(*x*_2*j*_(*t*)) and state *x*_1*i*_(*t*), *M*_*bji*_ is the connection memristor between the amplifier *f*_*j*_(*x*_2*j*_(*t*−σ(*t*))) and state *x*_1*i*_(*t*), *M*_*cij*_ is the connection memristor between the amplifier *g*_*i*_(*x*_1*i*_(*t*)) and state *x*_2*j*_(*t*), *M*_*dij*_ is the connection memristor between the amplifier *g*_*i*_(*x*_1*i*_(*t*−τ(*t*))) and state *x*_2*j*_(*t*), *R*_*ij*_ and *C*_*ij*_ are the resistor and capacitor.

**Figure 2:**
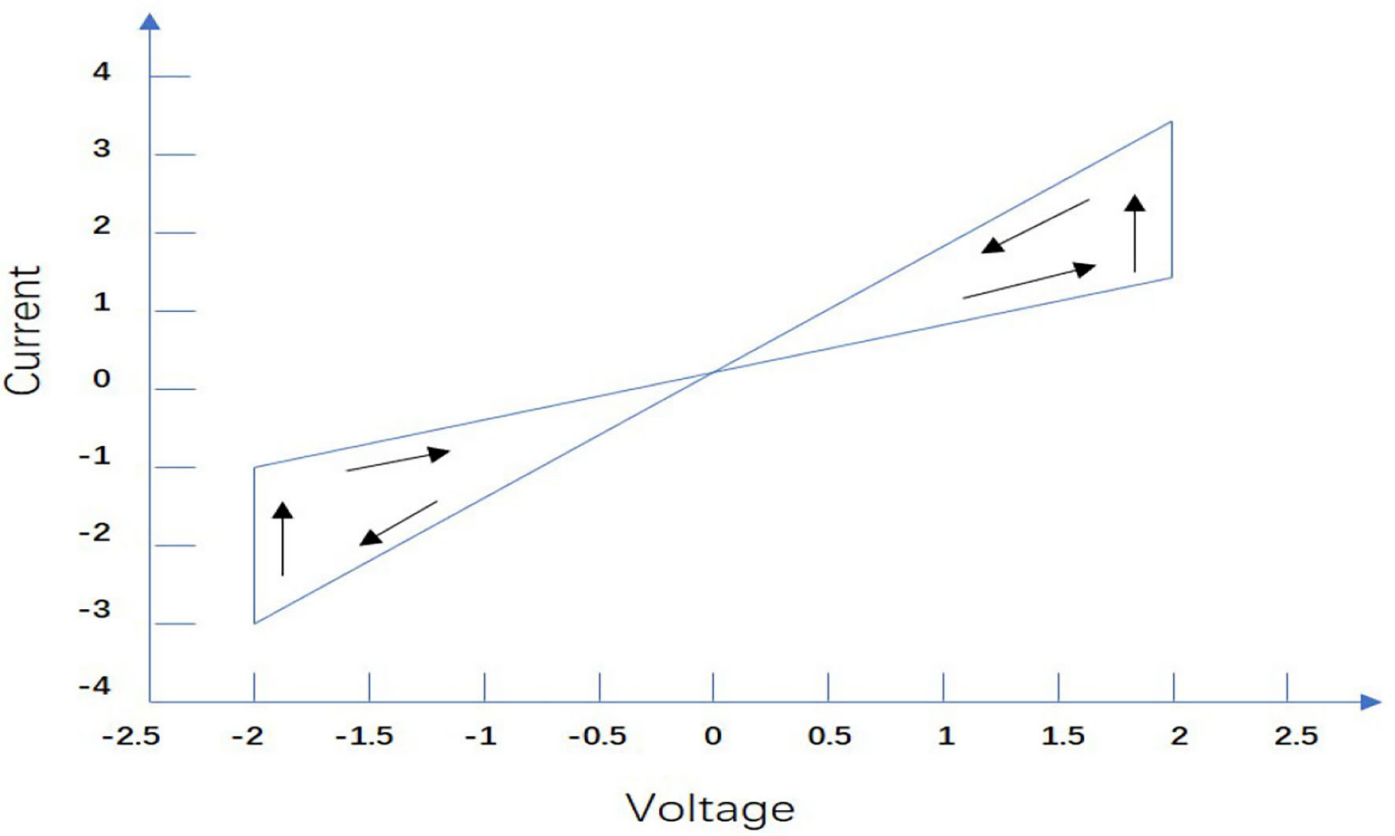
Typical current-voltage characteristic of a memristor.

Figure 2 illustrates the simplified current- voltage characteristics of the memristor, we define the neuron self-inhibition and connection weight as the following state correlation functions:


$$\begin{array}{l} \eta_{i}^{R}(x)=\{\begin{array}{l} {\hat{\eta }}_{i}^{R},|x|\;\geq T_{i},\\ {\overset{̬}{\eta }}_{i}^{R},|x|\;<T_{i}, \end{array}\;\eta_{i}^{I}(x)=\{\begin{array}{l} {\hat{\eta }}_{i}^{I},|x|\;\geq T_{i},\\ {\overset{̬}{\eta }}_{i}^{I},|x|\;<T_{i}, \end{array}\;\xi_{j}^{R}(x)=\{\begin{array}{l} {\hat{\xi }}_{j}^{R},|x|\;\geq T_{j}^{{\;}^{\prime }},\\ {\overset{̬}{\xi }}_{j}^{R},|x|\;<T_{j}^{{\;}^{\prime }}, \end{array}\\ \; \end{array}$$

$$\begin{array}{l} \xi_{j}^{I}(x)=\{\begin{array}{c} {\hat{\xi }}_{j}^{I},|x|\;\geq T_{j}^{{\;}^{\prime }},\\ {\overset{̬}{\xi }}_{j}^{I},|x|\;<T_{j}^{{\;}^{\prime }}, \end{array}\;a_{ji}^{R}(x)=\{\begin{array}{c} {Â}_{ji}^{R},|x|\;\geq {ℵ}_{i},\\ {Ă}_{ji}^{R},|x|\;<{ℵ}_{i}, \end{array}\;a_{ji}^{I}(x)=\{\begin{array}{c} {Â}_{ji}^{I},|x|\;\geq {ℵ}_{i},\\ {Ă}_{ji}^{I},|x|\;<{ℵ}_{i}, \end{array} \end{array}$$

$$\begin{array}{l} b_{ji}^{R}(x)=\{\begin{array}{l} {\hat{B}}_{ji}^{R},|\;x|\;\geq {ℵ}_{i}^{{\;}^{\prime }},\\ {\overset{̬}{B}}_{ji}^{R},|x|\;<{ℵ}_{i}^{{\;}^{\prime }}, \end{array}\;b_{ji}^{I}(x)=\{\begin{array}{l} {\hat{B}}_{ji}^{I},|x|\;\geq {ℵ}_{i}^{{\;}^{\prime }},\\ {\overset{̬}{B}}_{ji}^{I},|x|\;<{ℵ}_{i}^{{\;}^{\prime }}, \end{array}\;c_{ij}^{R}(x)=\{\begin{array}{l} {\hat{C}}_{ij}^{R},|x|\;\geq \varpi_{j},\\ {Č}_{ij}^{R},|x|\;<\varpi_{j}, \end{array} \end{array}$$

$$\begin{array}{l} c_{ij}^{I}(x)=\{\begin{array}{l} {\hat{C}}_{ij}^{I},|x|\;\geq \varpi_{j},\\ {Č}_{ij}^{I},|x|\;<\varpi_{j}, \end{array}\;d_{ij}^{R}(x)=\{\begin{array}{l} {\hat{D}}_{ij}^{R},|x|\;\geq \varpi_{j}^{{\;}^{\prime }},\\ {Ď}_{ij}^{R},|x|\;<\varpi_{j}^{{\;}^{\prime }}, \end{array}\;d_{ij}^{I}(x)=\{\begin{array}{l} {\hat{D}}_{ij}^{I},|x|\;\geq \varpi_{j}^{{\;}^{\prime }},\\ {Ď}_{ij}^{I},|x|\;<\varpi_{j}^{{\;}^{\prime }}, \end{array} \end{array}$$

where the switching jumps $T_{i},T_{j}^{{\;}^{\prime }},{ℵ}_{i},{ℵ}_{i}^{{\;}^{\prime }},\varpi_{j},\varpi_{j}^{{\;}^{\prime }}>0$.

**Figure 3:**
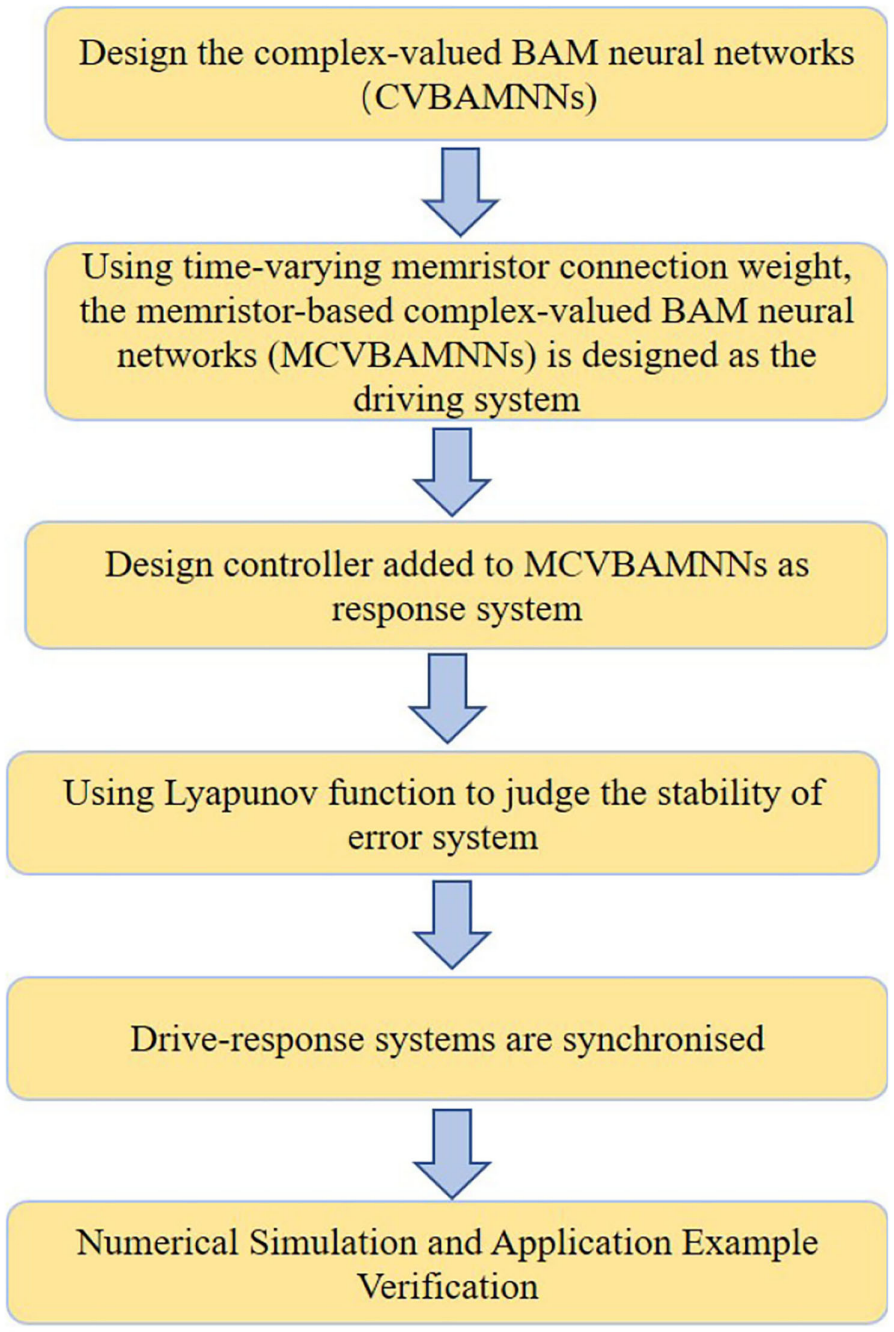
Flow chart of the system.

System (1) is called the drive system, the response system can be described as follows:


(2) $$\begin{array}{l} \{\begin{array}{c} {ẏ}_{1i}^{u}\left(t\right)=-\eta_{i}^{u}\left(y_{1i}^{u}\left(t-\tau \left(t\right)\right)\right)y_{1i}^{u}\left(t-\tau \left(t\right)\right)+\overset{m}{\underset{j=1}{\sum }}a_{ji}^{u}\left(y_{1i}^{u}\left(t\right)\right)f_{j}^{u}\left(y_{2j}^{u}\left(t\right)\right)\\ +\overset{m}{\underset{j=1}{\sum }}b_{ji}^{u}\left(y_{1i}^{u}\left(t-\tau \left(t\right)\right)\right)f_{j}^{u}\left(y_{2j}^{u}\left(t-\sigma \left(t\right)\right)\right)+u_{i}^{u}\left(t\right),\\ {ẏ}_{2j}^{u}\left(t\right)=-\xi_{j}^{u}\left(y_{2j}^{u}\left(t-\sigma \left(t\right)\right)\right)y_{2j}^{u}\left(t-\sigma \left(t\right)\right)+\overset{n}{\underset{i=1}{\sum }}c_{ij}^{u}\left(y_{2j}^{u}\left(t\right)\right)g_{i}^{u}\left(y_{1i}^{u}\left(t\right)\right)\\ +\overset{n}{\underset{i=1}{\sum }}d_{ij}^{u}\left(y_{2j}^{u}\left(t-\sigma \left(t\right)\right)\right)g_{i}^{u}\left(y_{1i}^{u}\left(t-\tau \left(t\right)\right)\right)+v_{j}^{u}\left(t\right), \end{array} \end{array}$$

where *i* = 1, 2, ..., *n*, *j* = 1, 2, ...*m*; $y_{1i}^{u}\left(t\right),y_{2j}^{u}\left(t\right)\in \mathbb{C}$. The initial values of the system (2) are $y_{1}^{u}\left(0\right)=\phi_{1}^{u}\left(s\right)$ and $y_{2}^{u}\left(0\right)=\phi_{2}^{u}\left(s\right)$. $u_{i}^{u}\left(t\right)$ and $v_{j}^{u}\left(t\right)$ are denoted as controllers. The remaining parameters are similar to those of the drive system. To better understand the following work, Figure 3 is the flow chart of the system.

** Definition 1**. Filippov (1999) consider dynamical systems with discontinuous right-hand side ẋ(*t*) = *F*(*t, x*), *x*(*t*) is a solution of the differential system on [0,T) in Filippov's sense, if *x*(*t*) is absolutely continuous and satisfies the differential inclusion of


$$\dot{x}\left(t\right)\in K\left[F\right]\left(t,x\right),$$

where


$$K\left[F\right]\left(t,x\right)=\underset{\delta >0}{⋂}\underset{\mu \left(N\right)=0}{⋂}\bar{co}\left[F\left(B\left(x,\delta \right)\backslash N\right),t\right],$$

where $\bar{co}\left[\cdot \right]$ is the convex closure hull of a set, *B*(*x*, δ) = {*y*:∥*y*−*x*∥ ≤ δ} is the ball centered at *x*(*t*) with radius δ, and μ(*N*) is the Lebesgue measure of set *N*.

** Definition 2**. Hu et al. (2017) the MCVBAMNNs (1) and (2) are said to achieve fixed-time synchronization if there exists a fixed-time *T*_*max*_>0, which is independent of the initial values but may be relevant with some parameters of MCVBAMNNs and controller, and a settling time function *T*(*e*(0)) ≤ *T*_*max*_ such that $\lim_{t\to T\left(e\left(0\right)\right)}∥e\left(t\right){∥}_{2}=0$ and ∥*e*(*t*)∥_2_ ≡ 0 for ∀*t* > *T*(*e*(0)).

** Definition 3**. Anguiano-Gijon et al. (2019) if the settling time *T*(*e*(0)) of fixed-time stability can be predicted by adjusting the constant *T*_*c*_, it means that the drive-response systems can achieve globally predefined-time stability, where *T*(*e*(0)) ≤ *T*_*c*_, ∀*e*(0) ∈ ℝ^*n*^.

** Remark 1**. Predefined-time synchronization is a special kind of fixed-time synchronization. The problem of predefined-time synchronization of drive-response systems can be converted into the predefined-time stability of the error systems. The purpose of this paper is to design an appropriate controller to stabilize the error systems in the expected time by adjusting the controller parameter *T*_*c*_.

Based on Definition 1 and the theory of differential inclusion, the drive system (1) can be written as


(3) $$\begin{array}{l} \{\begin{array}{l} {\dot{x}}_{1i}^{u}\left(t\right)=-\bar{co}\left(\eta_{i}^{u}\left(x_{1i}^{u}\left(t-\tau \left(t\right)\right)\right)\right)x_{1i}^{u}\left(t-\tau \left(t\right)\right)+\overset{m}{\underset{j=1}{\sum }}\bar{co}\left(a_{ji}^{u}\left(x_{1i}^{u}\left(t\right)\right)\right)f_{j}^{u}\left(x_{2j}^{u}\left(t\right)\right)\\ \;+\overset{m}{\underset{j=1}{\sum }}\bar{co}\left(b_{ji}^{u}\left(x_{1i}^{u}\left(t-\tau \left(t\right)\right)\right)\right)f_{j}^{u}\left(x_{2j}^{u}\left(t-\sigma \left(t\right)\right)\right),\\ {\dot{x}}_{2j}^{u}\left(t\right)=-\bar{co}\left(\xi_{j}^{u}\left(x_{2j}^{u}\left(t-\sigma \left(t\right)\right)\right)\right)x_{2j}^{u}\left(t-\sigma \left(t\right)\right)+\overset{n}{\underset{i=1}{\sum }}\bar{co}\left(c_{ij}^{u}\left(x_{2j}^{u}\left(t\right)\right)\right)g_{i}^{u}\left(x_{1i}^{u}\left(t\right)\right)\\ \;+\overset{n}{\underset{i=1}{\sum }}\bar{co}\left(d_{ij}^{u}\left(x_{2j}^{u}\left(t-\sigma \left(t\right)\right)\right)\right)g_{i}^{u}\left(x_{1i}^{u}\left(t-\tau \left(t\right)\right)\right). \end{array} \end{array}$$

The response system (2) is represented as


(4) $$\begin{array}{l} \{\begin{array}{l} {ẏ}_{1i}^{u}\left(t\right)=-\bar{co}\left(\eta_{i}^{u}\left(y_{1i}^{u}\left(t-\tau \left(t\right)\right)\right)\right)y_{1i}^{u}\left(t-\tau \left(t\right)\right)+\overset{m}{\underset{j=1}{\sum }}\bar{co}\left(a_{ji}^{u}\left(y_{1i}^{u}\left(t\right)\right)\right)f_{j}^{u}\left(y_{2j}^{u}\left(t\right)\right)\\ \;+\overset{m}{\underset{j=1}{\sum }}\bar{co}\left(b_{ji}^{u}\left(y_{1i}^{u}\left(t-\tau \left(t\right)\right)\right)\right)f_{j}^{u}\left(y_{2j}^{u}\left(t-\sigma \left(t\right)\right)\right)+u_{i}^{u}\left(t\right),\\ {ẏ}_{2j}^{u}\left(t\right)=-\bar{co}\left(\xi_{j}^{u}\left(y_{2j}^{u}\left(t-\sigma \left(t\right)\right)\right)\right)y_{2j}^{u}\left(t-\sigma \left(t\right)\right)+\overset{n}{\underset{i=1}{\sum }}\bar{co}\left(c_{ij}^{u}\left(y_{2j}^{u}\left(t\right)\right)\right)g_{i}^{u}\left(y_{1i}^{u}\left(t\right)\right)\\ \;+\overset{n}{\underset{i=1}{\sum }}\bar{co}\left(d_{ij}^{u}\left(y_{2j}^{u}\left(t-\sigma \left(t\right)\right)\right)\right)g_{i}^{u}\left(y_{1i}^{u}\left(t-\tau \left(t\right)\right)\right)+v_{j}^{u}\left(t\right). \end{array} \end{array}$$

To obtain the synchronization criteria by set-valued mapping, let


$$\begin{array}{l} {\tilde{\eta }}_{i}^{R}=max\{|{\hat{\eta }}_{i}^{R}|,|{\overset{ˇ}{\eta }}_{i}^{R}|\},\;{\tilde{\eta }}_{i}^{I}=max\{|{\hat{\eta }}_{i}^{I}|,|{\overset{ˇ}{\eta }}_{i}^{I}|\};\\ {\tilde{\xi }}_{j}^{R}=max\{|{\hat{\xi }}_{j}^{R}|,|{\overset{ˇ}{\xi }}_{j}^{R}|\},\;{\tilde{\xi }}_{j}^{I}=max\{|{\hat{\xi }}_{j}^{I}|,|{\overset{ˇ}{\xi }}_{j}^{I}|\};\\ {ã}_{ji}^{R}=max\{|{Â}_{ji}^{R}|,|{Ă}_{ji}^{R}|\},\;{ã}_{ji}^{I}=max\{|{Â}_{ji}^{I}|,|{Ă}_{ji}^{I}|\};\\ {\tilde{b}}_{ji}^{R}=max\{|{\hat{B}}_{ji}^{R}|,|{\overset{ˇ}{B}}_{ji}^{R}|\},\;{\tilde{b}}_{ji}^{I}=max\{|{\hat{B}}_{ji}^{I}|,|{\overset{ˇ}{B}}_{ji}^{I}|\};\\ {\tilde{c}}_{ij}^{R}=max\{|{\hat{C}}_{ij}^{R}|,|{Č}_{ij}^{R}|\},\;{\tilde{c}}_{ij}^{I}=max\{|{\hat{C}}_{ij}^{I}|,|{Č}_{ij}^{I}|\};\\ {\tilde{d}}_{ij}^{R}=max\{|{\hat{D}}_{ij}^{R}|,|{Ď}_{ij}^{R}|\},\;{\tilde{d}}_{ij}^{I}=max\{|{\hat{D}}_{ij}^{I}|,|{Ď}_{ij}^{I}|\}; \end{array}$$

The synchronization errors are defined as $e_{1i}^{u}\left(t\right)=y_{1i}^{u}\left(t\right)-x_{1i}^{u}\left(t\right)$, $e_{2j}^{u}\left(t\right)=y_{2j}^{u}\left(t\right)-x_{2j}^{u}\left(t\right)$, we can conclude that


(5) $$\begin{array}{l} \{\begin{array}{c} {\dot{e}}_{1i}^{R}\left(t\right)=P\left(t\right)+W_{i}^{R}\left(t\right)+u_{i}^{R}\left(t\right),\\ {\dot{e}}_{1i}^{I}\left(t\right)=\hat{P}\left(t\right)+W_{i}^{I}\left(t\right)+u_{i}^{I}\left(t\right),\\ {\dot{e}}_{2j}^{R}\left(t\right)=H\left(t\right)+Q_{j}^{R}\left(t\right)+v_{j}^{R}\left(t\right),\\ {\dot{e}}_{2j}^{I}\left(t\right)=\hat{H}\left(t\right)+Q_{j}^{I}\left(t\right)+v_{j}^{I}\left(t\right), \end{array} \end{array}$$

where *i* = 1, 2, ..., *n*, *j* = 1, 2, ..., *m*; the initial values of error system (5) are $e_{1}^{R}\left(s\right)=\phi_{1}^{R}\left(s\right)-\varphi_{1}^{R}\left(s\right)$, $e_{1}^{I}\left(s\right)=\phi_{1}^{I}\left(s\right)-\varphi_{1}^{I}\left(s\right)$, $e_{2}^{R}\left(s\right)=\phi_{2}^{R}\left(s\right)-\varphi_{2}^{R}\left(s\right)$, $e_{2}^{I}\left(s\right)=\phi_{2}^{I}\left(s\right)-\varphi_{2}^{I}\left(s\right)$. We define *P*(*t*), $\hat{P}\left(t\right)$, *H*(*t*), Ĥ(*t*), $W_{i}^{R}\left(t\right)$, $W_{i}^{I}\left(t\right)$, $Q_{j}^{R}\left(t\right)$, $Q_{j}^{I}\left(t\right)$ as follows:


(6) $$\begin{array}{l} P\left(t\right)=-\left[{\tilde{\eta }}_{i}^{R}y_{1i}^{R}\left(t-\tau \left(t\right)\right)-{\tilde{\eta }}_{i}^{I}y_{1i}^{I}\left(t-\tau \left(t\right)\right)\right]+\left[{\tilde{\eta }}_{i}^{R}x_{1i}^{R}\left(t-\tau \left(t\right)\right)-{\tilde{\eta }}_{i}^{I}x_{1i}^{I}\left(t-\tau \left(t\right)\right)\right], \end{array}$$

(7) $$\begin{array}{l} \hat{P}\left(t\right)=-\left[{\tilde{\eta }}_{i}^{R}y_{1i}^{I}\left(t-\tau \left(t\right)\right)+{\tilde{\eta }}_{i}^{I}y_{1i}^{R}\left(t-\tau \left(t\right)\right)\right]+\left[{\tilde{\eta }}_{i}^{R}x_{1i}^{I}\left(t-\tau \left(t\right)\right)+{\tilde{\eta }}_{i}^{I}x_{1i}^{R}\left(t-\tau \left(t\right)\right)\right], \end{array}$$

(8) $$\begin{array}{l} H\left(t\right)=-\left[{\tilde{\xi }}_{j}^{R}y_{2j}^{R}\left(t-\sigma \left(t\right)\right)-{\tilde{\xi }}_{j}^{I}y_{2j}^{I}\left(t-\sigma \left(t\right)\right)\right]+\left[{\tilde{\xi }}_{j}^{R}x_{2j}^{R}\left(t-\sigma \left(t\right)\right)-{\tilde{\xi }}_{j}^{I}x_{2j}^{I}\left(t-\sigma \left(t\right)\right)\right], \end{array}$$

(9) $$\begin{array}{l} \hat{H}\left(t\right)=-\left[{\tilde{\xi }}_{j}^{R}y_{2j}^{R}\left(t-\sigma \left(t\right)\right)+{\tilde{\xi }}_{j}^{I}y_{2j}^{R}\left(t-\sigma \left(t\right)\right)\right]+\left[{\tilde{\xi }}_{j}^{R}x_{2j}^{I}\left(t-\sigma \left(t\right)\right)+{\tilde{\xi }}_{j}^{I}x_{2j}^{R}\left(t-\sigma \left(t\right)\right)\right], \end{array}$$

(10) $$\begin{array}{l} \begin{array}{l} W_{i}^{R}\left(t\right)=\overset{m}{\underset{j=1}{\sum }}\left\{{ã}_{ji}^{R}f_{j}^{R}\left(y_{2j}^{R}\left(t\right)\right)-{ã}_{ji}^{R}f_{j}^{R}\left(x_{2j}^{R}\left(t\right)\right)+{ã}_{ji}^{I}f_{j}^{I}\left(x_{2j}^{I}\left(t\right)\right)-{ã}_{ji}^{I}f_{j}^{I}\left(y_{2j}^{I}\left(t\right)\right)\right\}\\ \;+\overset{m}{\underset{j=1}{\sum }}\left\{{\tilde{b}}_{ji}^{R}f_{j}^{R}\left(y_{2j}^{R}\left(t-\sigma \left(t\right)\right)\right)-{\tilde{b}}_{ji}^{R}f_{j}^{R}\left(x_{2j}^{R}\left(t-\sigma \left(t\right)\right)\right)\right\}\\ \;+\overset{m}{\underset{j=1}{\sum }}\left\{{\tilde{b}}_{ji}^{I}f_{j}^{I}\left(x_{2j}^{I}\left(t-\sigma \left(t\right)\right)\right)-{\tilde{b}}_{ji}^{I}f_{j}^{I}\left(y_{2j}^{I}\left(t-\sigma \left(t\right)\right)\right)\right\}, \end{array} \end{array}$$

(11) $$\begin{array}{l} \begin{array}{l} W_{i}^{I}\left(t\right)=\overset{m}{\underset{j=1}{\sum }}\left\{{ã}_{ji}^{R}f_{j}^{I}\left(y_{2j}^{I}\left(t\right)\right)-{ã}_{ji}^{R}f_{j}^{I}\left(x_{2j}^{I}\left(t\right)\right)+{ã}_{ji}^{I}f_{j}^{R}\left(y_{2j}^{R}\left(t\right)\right)-{ã}_{ji}^{I}f_{j}^{R}\left(x_{2j}^{R}\left(t\right)\right)\right\}\\ \;+\overset{m}{\underset{j=1}{\sum }}\left\{{\tilde{b}}_{ji}^{R}f_{j}^{I}\left(y_{2j}^{I}\left(t-\sigma \left(t\right)\right)\right)-{\tilde{b}}_{ji}^{R}f_{j}^{I}\left(x_{2j}^{I}\left(t-\sigma \left(t\right)\right)\right)\right\}\\ \;+\overset{m}{\underset{j=1}{\sum }}\left\{{\tilde{b}}_{ji}^{I}f_{j}^{R}\left(y_{2j}^{R}\left(t-\sigma \left(t\right)\right)\right)-{\tilde{b}}_{ji}^{I}f_{j}^{R}\left(x_{2j}^{R}\left(t-\sigma \left(t\right)\right)\right)\right\}, \end{array} \end{array}$$

(12) $$\begin{array}{l} \begin{array}{l} Q_{j}^{R}\left(t\right)=\overset{n}{\underset{i=1}{\sum }}\left\{{\tilde{c}}_{ij}^{R}g_{i}^{R}\left(y_{1i}^{R}\left(t\right)\right)-{\tilde{c}}_{ij}^{R}g_{i}^{R}\left(x_{1i}^{R}\left(t\right)\right)+{\tilde{c}}_{ij}^{I}g_{i}^{I}\left(x_{1i}^{I}\left(t\right)\right)-{\tilde{c}}_{ij}^{I}g_{i}^{I}\left(y_{1i}^{I}\left(t\right)\right)\right\}\\ \;+\overset{n}{\underset{i=1}{\sum }}\left\{{\tilde{d}}_{ij}^{R}g_{i}^{R}\left(y_{1i}^{R}\left(t-\tau \left(t\right)\right)\right)-{\tilde{d}}_{ij}^{I}g_{i}^{R}\left(x_{1i}^{I}\left(t-\tau \left(t\right)\right)\right)\right\}\\ \;-\overset{n}{\underset{i=1}{\sum }}\left\{{\tilde{d}}_{ij}^{I}g_{i}^{I}\left(y_{1i}^{I}\left(t-\tau \left(t\right)\right)\right)-{\tilde{d}}_{ij}^{I}g_{i}^{I}\left(x_{1i}^{I}\left(t-\tau \left(t\right)\right)\right)\right\}, \end{array} \end{array}$$

(13) $$\begin{array}{l} \begin{array}{l} Q_{j}^{I}\left(t\right)=\overset{n}{\underset{i=1}{\sum }}\left\{{\tilde{c}}_{ij}^{I}g_{i}^{R}\left(y_{1i}^{R}\left(t\right)\right)-{\tilde{c}}_{ij}^{I}g_{i}^{R}\left(x_{1i}^{R}\left(t\right)\right)+{\tilde{c}}_{ij}^{R}g_{i}^{I}\left(y_{1i}^{I}\left(t\right)\right)-{\tilde{c}}_{ij}^{R}g_{i}^{I}\left(x_{1i}^{I}\left(t\right)\right)\right\}\\ \;+\overset{n}{\underset{i=1}{\sum }}\left\{{\tilde{d}}_{ij}^{R}g_{i}^{I}\left(y_{1i}^{I}\left(t-\tau \left(t\right)\right)\right)-{\tilde{d}}_{ij}^{R}g_{i}^{I}\left(x_{1i}^{I}\left(t-\tau \left(t\right)\right)\right)\right\}\\ \;+\overset{n}{\underset{i=1}{\sum }}\left\{{\tilde{d}}_{ij}^{I}g_{i}^{R}\left(y_{1i}^{R}\left(t-\tau \left(t\right)\right)\right)-{\tilde{d}}_{ij}^{I}g_{i}^{R}\left(x_{1i}^{R}\left(t-\tau \left(t\right)\right)\right)\right\}. \end{array} \end{array}$$

** Assumption 1**. Suppose the activation functions satisfy $|f_{j}^{R}|\leq M_{j}^{R}$, $|f_{j}^{I}|\leq M_{j}^{I}$, $|g_{i}^{R}|\leq N_{i}^{R}$, $|g_{i}^{I}|\leq N_{i}^{I}$, for $M_{j}^{R},M_{j}^{I},N_{i}^{R},N_{i}^{I}$ are positive constants, *i* = 1, 2, ..., *n*, *j* = 1, 2..., *m*.

** Lemma 1**. Guo et al. (2020) the following inequality holds: $|W_{i}^{R}\left(t\right)|\leq \Lambda_{i}^{R}$, $|W_{i}^{I}\left(t\right)|\leq \Lambda_{i}^{I}$, $|Q_{j}^{R}\left(t\right)|\leq \Omega_{i}^{R}$, $|Q_{j}^{I}\left(t\right)|\leq \Omega_{i}^{I}$ for


$$\begin{array}{l} \Lambda_{i}^{R}=2\overset{m}{\underset{j=1}{\sum^{\text{​}}}}[M_{j}^{R}({ã}_{ji}^{R}+{\tilde{b}}_{ji}^{R})+M_{j}^{I}({ã}_{ji}^{I}+{\tilde{b}}_{ji}^{I})];\;\Lambda_{i}^{I}=2\overset{m}{\underset{j=1}{\sum^{\text{​}}}}[M_{j}^{R}({ã}_{ji}^{I}+{\tilde{b}}_{ji}^{I})+M_{j}^{I}({ã}_{ji}^{R}+{\tilde{b}}_{ji}^{R})];\;\Omega_{i}^{R}=2\overset{n}{\underset{i=1}{\sum^{\text{​}}}}[N_{i}^{R}({\tilde{c}}_{ij}^{R}+{\tilde{d}}_{ij}^{R})+N_{i}^{I}({\tilde{c}}_{ij}^{I}+{\tilde{d}}_{ij}^{I})];\;\\ \;\Omega_{i}^{I}=2\overset{n}{\underset{i=1}{\sum^{\text{​}}}}[N_{i}^{R}({\tilde{c}}_{ij}^{I}+{\tilde{d}}_{ij}^{I})+N_{i}^{I}({\tilde{c}}_{ij}^{R}+{\tilde{d}}_{ij}^{R})]. \end{array}$$

** Lemma 2**. Hardy et al. (1952) if α_1_, α_2_, ..., α_*n*_ ≥0, 0 < ρ ≤ 1, ζ > 1, then we have


$$\begin{array}{l} \sum_{i=1}^{n}\alpha_{i}^{\rho }\geq {\left(\sum_{i=1}^{n}\alpha_{i}\right)}^{\rho },\;\sum_{i=1}^{n}\alpha_{i}^{\zeta }\;\geq n^{1-\zeta }{\left(\sum_{i=1}^{n}\alpha_{i}\right)}^{\zeta }. \end{array}$$

** Lemma 3**. Chen et al. (2020) suppose the continuous and positive definite function *V*(*t*) satisfies the following two conditions:

*(i)* *V*(*t*) = 0 ⇔ *t* = 0;

*(ii)* Any solution *t* of system *V*(*t*) satisfies


$$\begin{array}{l} \dot{V}(t)\leq -aV^{\rho }(t)-bV^{\zeta }(t)-cV(t), \end{array}$$

for *a, b, c* > 0, 0 < ρ < 1, and ζ > 1. Then the origin of the system *V*(*t*) is fixed-time stable and the settling time is estimated by


$$\begin{array}{l} T_{max}^{1}=\frac{1}{c\left(1-\rho \right)}\ln\left(1+\frac{c}{a}\right)+\frac{1}{c\left(\zeta -1\right)}\ln\left(1+\frac{c}{b}\right). \end{array}$$

** Lemma 4**. Polyakov (2012) suppose the continuous and positive definite function *V*(*t*) satisfies the following two conditions:

*(i)* *V*(*t*) = 0 ⇔ *t* = 0;

*(ii)* Any solution *t* of system *V*(*t*) satisfies


$$\begin{array}{l} D^{+}V\left(t\right)\leq -aV^{\rho }\left(t\right)-bV^{\zeta }\left(t\right), \end{array}$$

for *a, b* > 0, 0 < ρ < 1, and ζ > 1, where *D*^+^*V*(*t*) denotes the upper right-hand Dini derivative of *V*(*t*). Then the origin of the system *V*(*t*) is fixed-time stable and the settling time is estimated by


$$\begin{array}{l} T_{max}^{2}=\frac{1}{a\left(1-\rho \right)}+\frac{1}{b\left(\zeta -1\right)}. \end{array}$$

** Lemma 5**. Hu et al. (2017) suppose the continuous and positive definite function *V*(*t*) satisfies the following two conditions:

*(i)* *V*(*t*) = 0 ⇔ *t* = 0;

*(ii)* Any solution *t* of system *V*(*t*) satisfies


$$\begin{array}{l} \overset{∙}{V}\left(t\right)\leq -aV^{\rho }\left(t\right)-bV^{\zeta }\left(t\right), \end{array}$$

for *a, b* > 0, 0 < ρ < 1, and ζ > 1. Then the origin of the system *V*(*t*) is fixed-time stable and the settling time is estimated by


$$\begin{array}{l} T_{max}^{3}=\frac{1}{a}\cdot {\left(\frac{a}{b}\right)}^{\frac{1-\rho }{\zeta -\rho }}\left(\frac{1}{1-\rho }+\frac{1}{\zeta -1}\right). \end{array}$$

** Lemma 6**. Parsegov et al. (2013) suppose the continuous and positive definite function *V*(*t*) satisfies the following two conditions:

*(i)* *V*(*t*) = 0 ⇔ *t* = 0;

*(ii)* Any solution *t* of system *V*(*t*) satisfies


$$\begin{array}{l} D^{+}V(t))\leq -aV^{\rho }(t))-bV^{\zeta }(t) \end{array}$$

for $a,b>0,\rho =1-\frac{1}{2d}$, and $\zeta =1+\frac{1}{2d}$, where *d* > 1. Then the origin of the system *V*(*t*) is fixed-time stable, and the settling time is estimated by


$$\begin{array}{l} T_{max}^{4}=\frac{\pi d}{\sqrt{ab}}. \end{array}$$

** Remark 2**. If *m* > 0, *ln*(1 + *m*) < *m*. Since $\frac{c}{a}>0$,$\frac{c}{b}>0$, we have $ln\left(1+\frac{c}{a}\right)<\frac{c}{a}$, $ln\left(1+\frac{c}{b}\right)<\frac{c}{b}$, then $\frac{1}{c\left(1-\rho \right)}\ln\left(1+\frac{c}{a}\right)+\frac{1}{c\left(\zeta -1\right)}\ln\left(1+\frac{c}{b}\right)<\frac{1}{a\left(1-\rho \right)}+\frac{1}{b\left(\zeta -1\right)}$. Therefore, Lemma 3 can give a more accurate *T*_*max*_ than Lemma 4.

** Remark 3**. Most of the designed controllers are discontinuous. To ensure the solution existence of the error system, the Dini derivative is introduced to guarantee continuity at breakpoints.

## 3. Main results

### 3.1. Fixed-time synchronization analysis

We design the following controller:


(14) $$\begin{array}{l} \{\begin{array}{l} \begin{array}{l} u_{i}^{R}(t)=-w_{i}sign(e_{1i}^{R}(t))-\lambda_{1i}^{R}(e_{1i}^{R}(t-\tau (t))-\lambda_{1i}^{I}(e_{1i}^{I}(t-\tau (t)))\\ \;-sign(e_{1i}^{R}(t))[\lambda_{2i}|e_{1i}^{R}(t)|+\lambda_{3i}|e_{1i}^{R}(t){|}^{\alpha }+\lambda_{4i}|e_{1i}^{R}(t){|}^{\beta }], \end{array}\\ \begin{array}{l} u_{i}^{I}(t)=-\;k_{i}sign(e_{1i}^{I}(t))-w_{1i}^{R}(e_{1i}^{R}(t-\tau (t))-w_{1i}^{I}(e_{1i}^{I}(t-\tau (t)))\\ \;-sign(e_{1i}^{I}(t)))[\lambda_{2i}|e_{1i}^{I}(t)|+\lambda_{3i}|e_{1i}^{I}(t){|}^{\alpha }+\lambda_{4i}|e_{1i}^{I}(t){|}^{\beta }], \end{array}\\ v_{j}^{R}(t)=-p_{j}sign(e_{2j}^{R}(t))-k_{1j}^{R}(e_{2j}^{R}(t-\sigma (t))-k_{1j}^{I}(e_{2j}^{I}(t-\sigma (t)))\\ \begin{array}{l} \;-sign(e_{2j}^{R}(t)))[k_{2j}|e_{2j}^{R}(t)|+k_{3j}|e_{2j}^{R}(t){|}^{\alpha }+k_{4j}|e_{2j}^{R}(t){|}^{\beta }],\\ v_{j}^{I}(t)=-\;q_{j}sign(e_{2j}^{I}(t))-p_{1j}^{R}(e_{2j}^{R}(t-\sigma (t))-p_{1j}^{I}(e_{2j}^{I}(t-\sigma (t)))\\ \;-sign(e_{2j}^{I}(t)))[k_{2j}|e_{2j}^{I}(t)|+k_{3j}|e_{2j}^{I}(t){|}^{\alpha }+\;k_{4j}|e_{2j}^{I}(t){|}^{\beta }], \end{array} \end{array} \end{array}$$

where *i* = 1, 2, ..., *n*, *j* = 1, 2, ..., *m*, and the constants *w*_*i*_, $\lambda_{1i}^{R}$, $\lambda_{1i}^{I}$, *k*_*i*_, $w_{1i}^{R}$, $w_{1i}^{I}$, *p*_*j*_, $k_{1j}^{R}$, $k_{1j}^{I}$, *q*_*j*_, $p_{ij}^{R}$, $p_{1j}^{I}$ should be determined later. Meanwhile, λ_2*i*_, λ_3*i*_, λ_4*i*_, *k*_2*j*_, *k*_3*j*_, *k*_4*j*_ are any positive constants, and 0 < α < 1, β > 1.

** Theorem 1**. If $\lambda_{1i}^{R}\geq {\tilde{\eta }}_{i}^{R}$, $\lambda_{1i}^{I}\geq {\tilde{\eta }}_{i}^{I}$, $w_{1i}^{R}\geq {\tilde{\eta }}_{i}^{R}$, $w_{1i}^{I}\geq {\tilde{\eta }}_{i}^{I}$, $k_{1j}^{R}\geq {\tilde{\xi }}_{i}^{R}$, $k_{1j}^{I}\geq {\tilde{\xi }}_{i}^{I}$, $p_{1j}^{R}\geq {\tilde{\xi }}_{i}^{R}$, $p_{1j}^{I}\geq {\tilde{\xi }}_{i}^{I}$, $w_{i}\geq \Lambda_{i}^{R}$, $k_{i}\geq \Lambda_{i}^{I}$, $p_{j}\geq \Omega_{i}^{R}$, $q_{j}\geq \Omega_{i}^{I}$, *i* = 1, 2, ..., *n*, *j* = 1, 2, ..., *m*, then systems (1) and (2) can achieve the fixed-time synchronization under the controller (14). Additionally, the settling time is $T_{max}^{1}=\frac{1}{c\left(1-\rho \right)}\ln\left(1+\frac{c}{a}\right)+\frac{1}{c\left(\zeta -1\right)}\ln\left(1+\frac{c}{b}\right)$, where $a=\lambda \cdot 2^{\frac{\alpha +1}{2}}$, $\rho =\frac{\alpha +1}{2}$, *b* = 2μ, $\zeta =\frac{1+\beta }{2}$, *c* = 2ω, $\lambda =min\left\{\underset{i}{min}\left(\lambda_{3i}\right),\underset{j}{min}\left(k_{3j}\right)\right\}$, $\mu =min\left\{\underset{i}{min}\left(\lambda_{4i}\right)\cdot n^{\frac{1-\beta }{2}},\underset{j}{min}\left(k_{4j}\right)\cdot m^{\frac{1-\beta }{2}}\right\}$, $\omega =min\left\{\underset{i}{min}\left(\lambda_{2i}\right),\underset{j}{min}\left(k_{2j}\right)\right\}$.

**Proof 1.** To prove this theorem, we construct the Lyapunov function as follows:


$$\begin{array}{l} V^{u}\left(t\right)=V_{1}^{u}\left(t\right)+V_{2}^{u}\left(t\right),\\ \;=V_{1}^{R}\left(t\right)+V_{1}^{I}\left(t\right)+V_{2}^{R}\left(t\right)+V_{2}^{I}\left(t\right), \end{array}$$

where $V_{1}^{R}\left(t\right)=\frac{1}{2}\overset{n}{\underset{i=1}{\sum }}{\left(e_{1i}^{R}\left(t\right)\right)}^{2}$, $V_{1}^{I}\left(t\right)=\frac{1}{2}\overset{n}{\underset{i=1}{\sum }}{\left(e_{1i}^{I}\left(t\right)\right)}^{2}$, $V_{2}^{R}\left(t\right)=\frac{1}{2}\overset{m}{\underset{j=1}{\sum }}{\left(e_{2j}^{R}\left(t\right)\right)}^{2}$, $V_{2}^{I}\left(t\right)=\frac{1}{2}\overset{m}{\underset{j=1}{\sum }}{\left(e_{2j}^{I}\left(t\right)\right)}^{2}$.

We calculate the derivative of $V_{1}^{R}\left(t\right)$:


$$\begin{array}{l} {\overset{∙}{V}}_{1}^{R}\left(t\right)=\overset{n}{\underset{i=1}{\sum }}e_{1i}^{R}\left(t\right){ė}_{1i}^{R}\left(t\right),\\ \;=\overset{n}{\underset{i=1}{\sum }}|e_{1i}^{R}\left(t\right)|sign\left(e_{1i}^{R}\left(t\right)\right)\left\{P\left(t\right)+W_{i}^{R}\left(t\right)+u_{i}^{R}\left(t\right)\right\},\\ \;\leq \overset{n}{\underset{i=1}{\sum }}\left\{{\tilde{\eta }}_{i}^{R}|e_{1i}^{R}\left(t\right)||e_{1i}^{R}\left(t-\tau \left(t\right)\right)|+{\tilde{\eta }}_{i}^{I}|e_{1i}^{R}\left(t\right)||e_{1i}^{I}\left(t-\tau \left(t\right)\right)|\right\}+\overset{n}{\underset{i=1}{\sum }}|e_{1i}^{R}\left(t\right)||W_{i}^{R}\left(t\right)|\\ \;+\overset{n}{\underset{i=1}{\sum }}|e_{1i}^{R}\left(t\right)|sign\left(e_{1i}^{R}\left(t\right)\right)\left\{-w_{i}sign\left(e_{1i}^{R}\left(t\right)\right)-\lambda_{1i}^{R}\left(e_{1i}^{R}\left(t-\tau \left(t\right)\right)\right)-\lambda_{1i}^{I}\left(e_{1i}^{I}\left(t-\tau \left(t\right)\right)\right)\right\}\\ \;+\overset{n}{\underset{i=1}{\sum }}|e_{1i}^{R}\left(t\right)|sign\left(e_{1i}^{R}\left(t\right)\right)\left\{-sign\left(e_{1i}^{R}\left(t\right)\right)\left[\lambda_{2i}|e_{1i}^{R}\left(t\right)|+\lambda_{3i}|e_{1i}^{R}\left(t\right){|}^{\alpha }+\lambda_{4i}|e_{1i}^{R}\left(t\right){|}^{\beta }\right]\right\}. \end{array}$$

According to Assumption 1 and Lemma 1, we have


$$\begin{array}{l} \begin{array}{l} {\overset{∙}{V}}_{1}^{R}\left(t\right)\leq -\overset{n}{\underset{i=1}{\sum }}\left[\lambda_{2i}|e_{1i}^{R}\left(t\right){|}^{2}+\lambda_{3i}|e_{1i}^{R}\left(t\right){|}^{\alpha +1}+\lambda_{4i}|e_{1i}^{R}\left(t\right){|}^{\beta +1}\right]+\overset{n}{\underset{i=1}{\sum }}\left(\Lambda_{i}^{R}-w_{i}\right)|e_{1i}^{R}\left(t\right)|\\ \;+\overset{n}{\underset{i=1}{\sum }}\left\{\left({\tilde{\eta }}_{i}^{R}-\lambda_{1i}^{R}\right)|e_{1i}^{R}\left(t\right)||e_{1i}^{R}\left(t-\tau \left(t\right)\right)|\right\}+\overset{n}{\underset{i=1}{\sum }}\left\{\left({\tilde{\eta }}_{i}^{I}-\lambda_{1i}^{I}\right)|e_{1i}^{R}\left(t\right)||e_{1i}^{I}\left(t-\tau \left(t\right)\right)|\right\}.\\ \;\leq -\overset{n}{\underset{i=1}{\sum }}\left[\lambda_{2i}|e_{1i}^{R}\left(t\right){|}^{2}+\lambda_{3i}|e_{1i}^{R}\left(t\right){|}^{\alpha +1}+\lambda_{4i}|e_{1i}^{R}\left(t\right){|}^{\beta +1}\right],\\ \;\leq -\underset{i}{min}\left(\lambda_{2i}\right)\overset{n}{\underset{i=1}{\sum }}|e_{1i}^{R}\left(t\right){|}^{2}-\underset{i}{min}\left(\lambda_{3i}\right)\overset{n}{\underset{i=1}{\sum }}|e_{1i}^{R}\left(t\right){|}^{\alpha +1}-\underset{i}{min}\left(\lambda_{4i}\right)\overset{n}{\underset{i=1}{\sum }}|e_{1i}^{R}\left(t\right){|}^{\beta +1},\\ \;\leq -\underset{i}{min}\left(\lambda_{2i}\right)\left(\overset{n}{\underset{i=1}{\sum }}|e_{1i}^{R}\left(t\right){|}^{2}\right)-\underset{i}{min}\left(\lambda_{3i}\right){\left(\overset{n}{\underset{i=1}{\sum }}|e_{1i}^{R}\left(t\right){|}^{2}\right)}^{\frac{\alpha +1}{2}}-n^{\frac{1-\beta }{2}}\cdot \underset{i}{min}\left(\lambda_{4i}\right){\left(\overset{n}{\underset{i=1}{\sum }}|e_{1i}^{R}\left(t\right){|}^{2}\right)}^{\frac{\beta +1}{2}},\\ \;\leq -2\underset{i}{min}\left(\lambda_{2i}\right)\cdot V_{1}^{R}\left(t\right)-2^{\frac{\alpha +1}{2}}\underset{i}{min}\left(\lambda_{3i}\right)\cdot {\left(V_{1}^{R}\left(t\right)\right)}^{\frac{\alpha +1}{2}}-n^{\frac{1-\beta }{2}}\cdot 2^{\frac{\beta +1}{2}}\underset{i}{min}\left(\lambda_{4i}\right)\cdot {\left(V_{1}^{R}\left(t\right)\right)}^{\frac{\beta +1}{2}}. \end{array} \end{array}$$

The proofs of $V_{1}^{I}\left(t\right)$, $V_{2}^{R}\left(t\right)$, and $V_{2}^{I}\left(t\right)$ are similar with that of $V_{1}^{R}\left(t\right)$:


$$\begin{array}{l} \begin{array}{c} {\overset{∙}{V}}_{1}^{I}\left(t\right)\leq -2\underset{i}{min}\left(\lambda_{2i}\right)\cdot V_{1}^{I}\left(t\right)-2^{\frac{\alpha +1}{2}}\underset{i}{min}\left(\lambda_{3i}\right)\cdot {\left(V_{1}^{I}\left(t\right)\right)}^{\frac{\alpha +1}{2}}\\ -n^{\frac{1-\beta }{2}}\cdot 2^{\frac{\beta +1}{2}}\underset{i}{min}\left(\lambda_{4i}\right)\cdot {\left(V_{1}^{I}\left(t\right)\right)}^{\frac{\beta +1}{2}},\\ {\overset{∙}{V}}_{2}^{R}\left(t\right)\leq -2\underset{j}{min}\left(k_{2j}\right)\cdot V_{2}^{R}\left(t\right)-2^{\frac{\alpha +1}{2}}\underset{j}{min}\left(k_{3j}\right)\cdot {\left(V_{2}^{R}\left(t\right)\right)}^{\frac{\alpha +1}{2}}\\ -m^{\frac{1-\beta }{2}}\cdot 2^{\frac{\beta +1}{2}}\underset{j}{min}\left(k_{4j}\right)\cdot {\left(V_{2}^{R}\left(t\right)\right)}^{\frac{\beta +1}{2}},\\ {\overset{∙}{V}}_{2}^{I}\left(t\right)\leq -2\underset{j}{min}\left(k_{2j}\right)\cdot V_{2}^{I}\left(t\right)-2^{\frac{\alpha +1}{2}}\underset{j}{min}\left(k_{3j}\right)\cdot {\left(V_{2}^{I}\left(t\right)\right)}^{\frac{\alpha +1}{2}}\\ -m^{\frac{1-\beta }{2}}\cdot 2^{\frac{\beta +1}{2}}\underset{j}{min}\left(k_{4j}\right)\cdot {\left(V_{2}^{I}\left(t\right)\right)}^{\frac{\beta +1}{2}}. \end{array} \end{array}$$

According to the analysis above, we can obtain that


$$\begin{array}{l} {\dot{V}}^{u}(t)\leq -2\cdot \{\underset{i}{min}(\lambda_{2i})(V_{1}^{R}(t))+\underset{i}{min}(\lambda_{2i})(V_{1}^{I}(t))\\ \;+\underset{j}{min}(k_{2j})(V_{2}^{R}(t))+\underset{j}{min}(k_{2j})(V_{2}^{I}(t))\}\\ \;-2^{\frac{\alpha +1}{2}}\cdot \{\underset{i}{min}(\lambda_{3i}){\left(V_{1}^{R}(t)\right)}^{\frac{\alpha +1}{2}}+\underset{i}{min}(\lambda_{3i}){\left(V_{1}^{I}(t)\right)}^{\frac{\alpha +1}{2}}\\ \;+\underset{j}{min}(k_{3j}){\left(V_{2}^{R}(t)\right)}^{\frac{\alpha +1}{2}}\text{​}+\text{​}\underset{j}{\;min}(k_{3j}){\left(V_{2}^{I}(t)\right)}^{\frac{\alpha +1}{2}}\}\\ \;-2^{\frac{\beta +1}{2}}\cdot \{n^{\frac{1-\beta }{2}}\cdot \underset{i}{\;min}(\lambda_{4i}){\left(V_{1}^{R}(t)\right)}^{\frac{\beta +1}{2}}\text{​}+\text{ ​}n^{\frac{1-\beta }{2}}\cdot \underset{i}{\;min}(\lambda_{4i}){\left(V_{2}^{I}(t)\right)}^{\frac{\beta +1}{2}}\\ \;+m^{\frac{1-\beta }{2}}\cdot \;\underset{j}{min}(k_{4j}){\left(V_{2}^{R}(t)\right)}^{\frac{\beta +1}{2}}+m^{\frac{1-\beta }{2}}\cdot \;\underset{j}{min}(k_{4j}){\left(V_{2}^{I}(t)\right)}^{\frac{\beta +1}{2}}\}. \end{array}$$

Therefore, we get


$$\begin{array}{l} {\dot{V}}^{u}(t)\leq -2\;\cdot \{\underset{i}{min}(\lambda_{2i})\cdot [V_{1}^{R}(t)\text{​ }+\text{​ }V_{1}^{I}(t)]+\underset{j}{min}(k_{2j})\cdot \;[V_{1}^{R}(t)\text{​ }+\text{​ }V_{1}^{I}(t)]\}\\ \;-2^{\frac{\alpha +1}{2}}\cdot \{\underset{i}{min}(\lambda_{3i}){\left[V_{1}^{R}(t)\text{​ }+\text{​ }V_{1}^{I}(t)\right]}^{\frac{\alpha +1}{2}}+\underset{j}{min}(k_{3j}){\left[V_{2}^{R}(t)\text{​ }+\text{​ }V_{2}^{I}(t)\right]}^{\frac{\alpha +1}{2}}\}\;\\ \;-2^{\frac{\beta +1}{2}}\cdot \{n^{\frac{1-\beta }{2}}\cdot \;\underset{i}{min}(\lambda_{4i})\cdot {\left[V_{1}^{R}(t)\text{​ }+\text{​ }V_{1}^{I}(t)\right]}^{\frac{\beta +1}{2}}+m^{\frac{1-\beta }{2}}\cdot \underset{j}{\;min}(k_{4j})\cdot \;{\left[V_{2}^{R}(t)\text{​ }+\text{ ​}V_{2}^{I}(t)\right]}^{\frac{\beta +1}{2}}\},\\ \;\leq -2^{\frac{\alpha +1}{2}}\lambda \cdot \;[{\left(V_{1}^{u}(t)\right)}^{\frac{1+\alpha }{2}}+{\left(V_{2}^{u}(t)\right)}^{\frac{1+\alpha }{2}}]-2^{\frac{\beta +1}{2}}\mu \cdot [{\left(V_{1}^{u}(t)\right)}^{\frac{1+\beta }{2}}+{\left(V_{2}^{u}(t)\right)}^{\frac{1+\beta }{2}}]\\ \;-2\omega \cdot [V_{1}^{u}(t)+V_{2}^{u}(t)],\\ \;\leq -2^{\frac{\alpha +1}{2}}\lambda \cdot {\left(V^{u}(t)\right)}^{\frac{1+\alpha }{2}}\text{​}-\text{​}2\mu \;\cdot {\left(V^{u}(t)\right)}^{\frac{1+\beta }{2}}\text{​}-\text{​}2\omega \;\cdot \;V^{u}(t). \end{array}$$

According to the above results and referring to Lemma 3, it can be obtained that the systems (1) and (2) have achieved fixed-time synchronization under controller (14). Then the settling time $T_{max}^{1}=\frac{1}{c\left(1-\rho \right)}\ln\left(1+\frac{c}{a}\right)+\frac{1}{c\left(\zeta -1\right)}\ln\left(1+\frac{c}{b}\right)$, and $a=\lambda \cdot 2^{\frac{\alpha +1}{2}}$, $\rho =\frac{\alpha +1}{2}$, *b* = 2μ, $\zeta =\frac{1+\beta }{2}$, *c* = 2ω, where $\lambda =min\left\{\underset{i}{min}\left(\lambda_{3i}\right),\underset{j}{min}\left(k_{3j}\right)\right\}$, $\mu =min\left\{\underset{i}{min}\left(\lambda_{4i}\right)\cdot n^{\frac{1-\beta }{2}},\underset{j}{min}\left(k_{4j}\right)\cdot m^{\frac{1-\beta }{2}}\right\}$, $\omega =min\left\{\underset{i}{min}\left(\lambda_{2i}\right),\underset{j}{min}\left(k_{2j}\right)\right\}$.

** Corollary 1**. If $\lambda_{1i}^{R}\geq {\tilde{\eta }}_{i}^{R}$, $\lambda_{1i}^{I}\geq {\tilde{\eta }}_{i}^{I}$, $w_{1i}^{R}\geq {\tilde{\eta }}_{i}^{R}$, $w_{1i}^{I}\geq {\tilde{\eta }}_{i}^{I}$, $k_{1j}^{R}\geq {\tilde{\xi }}_{i}^{R}$, $k_{1j}^{I}\geq {\tilde{\xi }}_{i}^{I}$, $p_{1j}^{R}\geq {\tilde{\xi }}_{i}^{R}$, $p_{1j}^{I}\geq {\tilde{\xi }}_{i}^{I}$, $w_{i}\geq \Lambda_{i}^{R}$, $k_{i}\geq \Lambda_{i}^{I}$, $p_{j}\geq \Omega_{i}^{R}$, $q_{j}\geq \Omega_{i}^{I}$, *i* = 1, 2, ..., *n*, *j* = 1, 2, ..., *m*, then the systems (1) and (2) can achieve fixed-time synchronization under the controller (14). Furthermore, $T_{max}^{2}=\frac{1}{a\left(1-\rho \right)}+\frac{1}{b\left(\zeta -1\right)}$, where $a=\lambda \cdot 2^{\frac{\alpha +1}{2}}$, $\rho =\frac{\alpha +1}{2}$, *b* = 2μ, $\zeta =\frac{1+\beta }{2}$.

**Proof 2.** Similarly, it can be proved that


$$\begin{array}{l} {\overset{∙}{V}}^{u}\left(t\right)\leq -a{\left(V^{u}\left(t\right)\right)}^{\frac{1+\alpha }{2}}-b{\left(V^{u}\left(t\right)\right)}^{\frac{1+\beta }{2}}-cV^{u}\left(t\right), \end{array}$$

where $a=2^{\frac{\alpha +1}{2}}\lambda$, *b* = 2μ, *c* = 2ω, $\lambda =min\left\{\underset{i}{min}\left(\lambda_{3i}\right),\underset{j}{min}\left(k_{3j}\right)\right\}$, $\mu =min\left\{\underset{i}{min}\left(\lambda_{4i}\right)\cdot n^{\frac{1-\beta }{2}},\underset{j}{min}\left(k_{4j}\right)\cdot m^{\frac{1-\beta }{2}}\right\}$. Since the Lyapunov function *V*^*u*^(*t*) is derivable, we have $D^{+}V^{u}\left(t\right)={\overset{∙}{V}}^{u}\left(t\right)$. Therefore,


$$\begin{array}{l} \begin{array}{c} D^{+}V^{u}\left(t\right)\leq -a{\left(V^{u}\left(t\right)\right)}^{\frac{1+\alpha }{2}}-b{\left(V^{u}\left(t\right)\right)}^{\frac{1+\beta }{2}}-cV^{u}\left(t\right),\\ \leq -a{\left(V^{u}\left(t\right)\right)}^{\frac{1+\alpha }{2}}-b{\left(V^{u}\left(t\right)\right)}^{\frac{1+\beta }{2}}. \end{array} \end{array}$$

According to Lemma 4, the origin of system (5) can achieve fixed-time stability.

** Corollary 2**. If $\lambda_{1i}^{R}\geq {\tilde{\eta }}_{i}^{R}$, $\lambda_{1i}^{I}\geq {\tilde{\eta }}_{i}^{I}$, $w_{1i}^{R}\geq {\tilde{\eta }}_{i}^{R}$, $w_{1i}^{I}\geq {\tilde{\eta }}_{i}^{I}$, $k_{1j}^{R}\geq {\tilde{\xi }}_{i}^{R}$, $k_{1j}^{I}\geq {\tilde{\xi }}_{i}^{I}$, $p_{1j}^{R}\geq {\tilde{\xi }}_{i}^{R}$, $p_{1j}^{I}\geq {\tilde{\xi }}_{i}^{I}$, $w_{i}\geq \Lambda_{i}^{R}$, $k_{i}\geq \Lambda_{i}^{I}$, $p_{j}\geq \Omega_{i}^{R}$, $q_{j}\geq \Omega_{i}^{I}$, *i* = 1, 2, ..., *n*, *j* = 1, 2, ..., *m*. According to Lemma 5, systems (1) and (2) can achieve fixed-time synchronization under the controller (14). Furthermore, $T_{max}^{3}=\frac{1}{a}\cdot {\left(\frac{a}{b}\right)}^{\frac{1-\rho }{\zeta -\rho }}\left(\frac{1}{1-\rho }+\frac{1}{\zeta -1}\right)$. where $a=\lambda \cdot 2^{\frac{\alpha +1}{2}}$, $\rho =\frac{\alpha +1}{2}$, *b* = 2μ, $\zeta =\frac{1+\beta }{2}$, $\lambda =min\left\{\underset{i}{min}\left(\lambda_{3i}\right),\underset{j}{min}\left(k_{3j}\right)\right\}$, $\mu =min\left\{\underset{i}{min}\left(\lambda_{4i}\right)\cdot n^{\frac{1-\beta }{2}},\underset{j}{min}\left(k_{4j}\right)\cdot m^{\frac{1-\beta }{2}}\right\}$. The proof process is similar to Corollary 1, so it is omitted here.

** Corollary 3**. If $\lambda_{1i}^{R}\geq {\tilde{\eta }}_{i}^{R}$, $\lambda_{1i}^{I}\geq {\tilde{\eta }}_{i}^{I}$, $w_{1i}^{R}\geq {\tilde{\eta }}_{i}^{R}$, $w_{1i}^{I}\geq {\tilde{\eta }}_{i}^{I}$, $k_{1j}^{R}\geq {\tilde{\xi }}_{i}^{R}$, $k_{1j}^{I}\geq {\tilde{\xi }}_{i}^{I}$, $p_{1j}^{R}\geq {\tilde{\xi }}_{i}^{R}$, $p_{1j}^{I}\geq {\tilde{\xi }}_{i}^{I}$, $w_{i}\geq \Lambda_{i}^{R}$, $k_{i}\geq \Lambda_{i}^{I}$, $p_{j}\geq \Omega_{i}^{R}$, $q_{j}\geq \Omega_{i}^{I}$, *i* = 1, 2, ..., *n*, *j* = 1, 2, ..., *m*. According to Lemma 6, systems (1) and (2) can achieve fixed-time synchronization under the controller (14). Furthermore, $T_{max}^{4}=\frac{\pi d}{\sqrt{ab}}.$ where $a=\lambda \cdot 2^{\frac{\alpha +1}{2}}$, *b* = 2μ, $\lambda =min\left\{\underset{i}{min}\left(\lambda_{3i}\right),\underset{j}{min}\left(k_{3j}\right)\right\}$, $\mu =min\left\{\underset{i}{min}\left(\lambda_{4i}\right)\cdot n^{\frac{1-\beta }{2}},\underset{j}{min}\left(k_{4j}\right)\cdot m^{\frac{1-\beta }{2}}\right\}$. The proof process is similar to Corollary 1, so it is omitted here.

### 3.2. Predefined-time synchronization analysis

** Theorem 2**. For system (5), if there exists a continuous and positive definite function *V*(*e*(*t*)):ℂ^*n*^ → ℝ, *T*_*c*_ is a user-defined parameter, and the following conditions hold:

*(i)* *V*(*e*(*t*)) = 0 ⇔ *e*(*t*) = 0;

*(ii)* For any *V*(*e*(*t*)) > 0, there exist *a, b, c* > 0, 0 < *p* < 1, *q* > 1 satisfying


$$\begin{array}{l} \overset{∙}{V}\left(e\left(t\right)\right)\leq -\frac{G_{c}}{T_{c}}\left(aV^{p}\left(e\left(t\right)\right)+bV^{q}\left(e\left(t\right)\right)+cV\left(e\left(t\right)\right)\right), \end{array}$$

then the origin of system (5) is predefined-time stable within predefined time *T*_*c*_, in which


$$\begin{array}{l} G_{c}=\frac{1}{c\left(1-p\right)}ln\left(1+\frac{c}{a}\right)+\frac{1}{c\left(q-1\right)}ln\left(1+\frac{c}{b}\right). \end{array}$$

**Proof 3.** For any *V*(*e*(*t*)) > 0, the corresponding analysis is shown as follows:


$$\begin{array}{l} \overset{∙}{V}\left(e\left(t\right)\right)\leq -\frac{G_{c}}{T_{c}}\left(aV^{p}\left(e\left(t\right)\right)+bV^{q}\left(e\left(t\right)\right)+cV\left(e\left(t\right)\right)\right). \end{array}$$

The setting time function is given as follows:


$$\begin{array}{l} T\left(e\left(0\right)\right)=\int_{0}^{T\left(e\left(0\right)\right)}dt. \end{array}$$

Then we have


$$\begin{array}{l} \begin{array}{l} T\left(e\left(0\right)\right)=\int_{0}^{T\left(e\left(0\right)\right)}dt,\\ \;\leq \int_{0}^{V\left(e\left(0\right)\right)}\frac{T_{c}}{G_{c}}\frac{1}{aV^{p}+bV^{q}+cV}dV,\\ \;\leq \int_{0}^{1}\frac{T_{c}}{G_{c}}\frac{1}{aV^{p}+bV^{q}+cV}dV+\int_{1}^{+\infty }\frac{T_{c}}{G_{c}}\frac{1}{aV^{p}+bV^{q}+cV}dV. \end{array} \end{array}$$

Let *W* = *V*^1−*p*^, *dW* = (1−*p*)*V*^−*p*^*dV*, $V=W^{\frac{1}{1-p}}$, then we have


$$\begin{array}{l} \int_{0}^{1}\frac{T_{c}}{G_{c}}\frac{1}{aV^{p}+bV^{q}+cV}dV\leq \int_{0}^{1}\frac{T_{c}}{G_{c}}\frac{1}{aV^{p}+cV}dV,\\ \;=\int_{0}^{1}\frac{T_{c}}{G_{c}}\frac{1}{1-p}\frac{V^{p}}{aV^{p}+cV}dW,\\ \;=\int_{0}^{1}\frac{T_{c}}{G_{c}}\frac{1}{1-p}\frac{1}{a+cW}dW,\\ \;=\frac{T_{c}}{G_{c}}\frac{1}{c\left(1-p\right)}ln\left(1+\frac{c}{a}\right). \end{array}$$

Let *Z* = *V*^1−*q*^, *dZ* = (1−*q*)*V*^−*q*^*dV*, $V=Z^{\frac{1}{1-q}}$, then we have


$$\begin{array}{l} \int_{1}^{+\infty }\frac{T_{c}}{G_{c}}\frac{1}{aV^{p}+bV^{q}+cV}dV\leq \int_{1}^{+\infty }\frac{T_{c}}{G_{c}}\frac{1}{bV^{q}+cV}dV,\\ \;=\int_{0}^{1}\frac{T_{c}}{G_{c}}\frac{1}{q-1}\frac{V^{q}}{bV^{q}+cV}dZ,\\ \;=\int_{0}^{1}\frac{T_{c}}{G_{c}}\frac{1}{q-1}\frac{1}{b+cZ}dZ,\\ \;=\frac{T_{c}}{G_{c}}\frac{1}{c\left(q-1\right)}ln\left(1+\frac{c}{b}\right). \end{array}$$

Therefore, we have


$$\begin{array}{l} T\left(e\left(0\right)\right)\leq \frac{T_{c}}{G_{c}}\left(\frac{1}{c\left(1-p\right)}ln\left(1+\frac{c}{a}\right)+\frac{1}{c\left(q-1\right)}ln\left(1+\frac{c}{b}\right)\right),\\ \;\leq T_{c}. \end{array}$$

In order to realize the predefined-time synchronization of systems (1) and (2), we designed the following controller:


(15) $$\begin{array}{l} \{\begin{array}{l} \begin{array}{l} u_{i}^{R}(t)=-w_{i}sign(e_{1i}^{R}(t))-\lambda_{1i}^{R}(e_{1i}^{R}(t-\tau (t))-\lambda_{1i}^{I}(e_{1i}^{I}(t-\tau (t)))\\ \;-sign(e_{1i}^{R}(t))\frac{G_{c}}{T_{c}}[\lambda_{2i}|e_{1i}^{R}(t)|+\lambda_{3i}|e_{1i}^{R}(t){|}^{\alpha }+\lambda_{4i}|e_{1i}^{R}(t){|}^{\beta }], \end{array}\\ \begin{array}{l} u_{i}^{I}(t)=-k_{i}sign(e_{1i}^{I}(t))-w_{1i}^{R}(e_{1i}^{R}(t-\tau (t))-w_{1i}^{I}(e_{1i}^{I}(t-\tau (t)))\\ \;-sign(e_{1i}^{I}(t)))\frac{G_{c}}{T_{c}}[\lambda_{2i}|e_{1i}^{I}(t)|+\lambda_{3i}|e_{1i}^{I}(t){|}^{\alpha }+\lambda_{4i}|e_{1i}^{I}(t){|}^{\beta }], \end{array}\\ v_{j}^{R}(t)=-p_{j}sign(e_{2j}^{R}(t))-k_{1j}^{R}(e_{2j}^{R}(t-\sigma (t))-k_{1j}^{I}(e_{2j}^{I}(t-\sigma (t)))\\ \begin{array}{l} \;-sign(e_{2j}^{R}(t)))\frac{G_{c}}{T_{c}}[k_{2j}|e_{2j}^{R}(t)|+k_{3j}|e_{2j}^{R}(t){|}^{\alpha }+k_{4j}|e_{2j}^{R}(t){|}^{\beta }],\\ v_{j}^{I}(t)=-q_{j}sign(e_{2j}^{I}(t))-p_{1j}^{R}(e_{2j}^{R}(t-\sigma (t))-p_{1j}^{I}(e_{2j}^{I}(t-\sigma (t)))\\ \;-sign(e_{2j}^{I}(t)))\frac{G_{c}}{T_{c}}[k_{2j}|e_{2j}^{I}(t)|+k_{3j}|e_{2j}^{I}(t){|}^{\alpha }+k_{4j}|e_{2j}^{I}(t){|}^{\beta }]. \end{array} \end{array} \end{array}$$

** Theorem 3**. If $\lambda_{1i}^{R}\geq {\tilde{\eta }}_{i}^{R}$, $\lambda_{1i}^{I}\geq {\tilde{\eta }}_{i}^{I}$, $w_{1i}^{R}\geq {\tilde{\eta }}_{i}^{R}$, $w_{1i}^{I}\geq {\tilde{\eta }}_{i}^{I}$, $k_{1j}^{R}\geq {\tilde{\xi }}_{i}^{R}$, $k_{1j}^{I}\geq {\tilde{\xi }}_{i}^{I}$, $p_{1j}^{R}\geq {\tilde{\xi }}_{i}^{R}$, $p_{1j}^{I}\geq {\tilde{\xi }}_{i}^{I}$, $w_{i}\geq \Lambda_{i}^{R}$, $k_{i}\geq \Lambda_{i}^{I}$, $p_{j}\geq \Omega_{i}^{R}$, $q_{j}\geq \Omega_{i}^{I}$, *i* = 1, 2, ..., *n*, *j* = 1, 2, ..., *m*, $G_{c}=\frac{1}{c\left(1-\rho \right)}ln\left(1+\frac{c}{a}\right)+\frac{1}{c\left(\xi -1\right)}ln\left(1+\frac{c}{b}\right)$, systems (1) and (2) can achieve the predefined-time synchronization within predefined time *T*_*c*_ and the controller (15), where $a=\lambda \cdot 2^{\frac{\alpha +1}{2}}$, *b* = 2μ, *c* = 2ω, $\rho =\frac{\alpha +1}{2}$, $\zeta =\frac{1+\beta }{2}$, $\lambda =min\left\{\underset{i}{min}\left(\lambda_{3i}\right),\underset{j}{min}\left(k_{3j}\right)\right\}$, $\mu =min\left\{\underset{i}{min}\left(\lambda_{4i}\right)\cdot n^{\frac{1-\beta }{2}},\underset{j}{min}\left(k_{4j}\right)\cdot m^{\frac{1-\beta }{2}}\right\}$, $\omega =min\left\{\underset{i}{min}\left(\lambda_{2i}\right),\underset{j}{min}\left(k_{2j}\right)\right\}$.

**Proof 4.** To prove this theorem, we construct the Lyapunov function as follows:


$$\begin{array}{l} V^{u}\left(t\right)=V_{1}^{u}\left(t\right)+V_{2}^{u}\left(t\right),\\ \;=V_{1}^{R}\left(t\right)+V_{1}^{I}\left(t\right)+V_{2}^{R}\left(t\right)+V_{2}^{I}\left(t\right), \end{array}$$

where $V_{1}^{R}\left(t\right)=\frac{1}{2}\overset{n}{\underset{i=1}{\sum }}{\left(e_{1i}^{R}\left(t\right)\right)}^{2}$, $V_{1}^{I}\left(t\right)=\frac{1}{2}\overset{n}{\underset{i=1}{\sum }}{\left(e_{1i}^{I}\left(t\right)\right)}^{2}$, $V_{2}^{R}\left(t\right)=\frac{1}{2}\overset{m}{\underset{j=1}{\sum }}{\left(e_{2j}^{R}\left(t\right)\right)}^{2}$, $V_{2}^{I}\left(t\right)=\frac{1}{2}\overset{m}{\underset{j=1}{\sum }}{\left(e_{2j}^{I}\left(t\right)\right)}^{2}$.

We calculate the derivative of $V_{1}^{R}\left(t\right)$ as follows:


$$\begin{array}{l} \begin{array}{l} {\dot{V}}_{1}^{R}(t)=\overset{n}{\underset{i=1}{\sum }}e_{1i}^{R}(t){\dot{e}}_{1i}^{R}(t),\\ \;=\overset{n}{\underset{i=1}{\sum }}|e_{1i}^{R}(t)|sign(e_{1i}^{R}(t))\{P(t)+W_{i}^{R}(t)+u_{i}^{R}(t)\},\\ \;\leq \overset{n}{\underset{i=1}{\sum }}\{{\tilde{\eta }}_{i}^{R}|e_{1i}^{R}(t)||e_{1i}^{R}(t\text{​}-\text{​}\tau (t))|\text{​ }+\text{ ​}{\tilde{\eta }}_{i}^{I}|e_{1i}^{R}(t)||e_{1i}^{I}(t\text{​}-\text{​}\tau (t))|\}+\overset{n}{\underset{i=1}{\sum }}|e_{1i}^{R}(t)||W_{i}^{R}(t)|\\ \;+\overset{n}{\underset{i=1}{\sum }}|e_{1i}^{R}(t)|sign(e_{1i}^{R}(t))\{-w_{i}sign(e_{1i}^{R}(t))-\lambda_{1i}^{R}(e_{1i}^{R}(t-\tau (t))-\lambda_{1i}^{I}(e_{1i}^{I}(t-\tau (t)))\}\\ \;+\overset{n}{\underset{i=1}{\sum }}|e_{1i}^{R}(t)|sign(e_{1i}^{R}(t))\{-sign(e_{1i}^{R}(t))\frac{Gc}{T_{c}}[\lambda_{2i}|e_{1i}^{R}(t)|+\lambda_{3i}|e_{1i}^{R}(t){|}^{\alpha }+\lambda_{4i}|e_{1i}^{R}(t){|}^{\beta }]\}. \end{array} \end{array}$$

According to Assumption 1 and Lemma 1, we have


$$\begin{array}{l} \begin{array}{l} {\overset{∙}{V}}_{1}^{R}\left(t\right)\leq -\overset{n}{\underset{i=1}{\sum }}\frac{G_{c}}{T_{c}}\left[\lambda_{2i}|e_{1i}^{R}\left(t\right){|}^{2}+\lambda_{3i}|e_{1i}^{R}\left(t\right){|}^{\alpha +1}+\lambda_{4i}|e_{1i}^{R}\left(t\right){|}^{\beta +1}\right]+\overset{n}{\underset{i=1}{\sum }}\left(\Lambda_{i}^{R}-w_{i}\right)|e_{1i}^{R}\left(t\right)|\\ \;+\overset{n}{\underset{i=1}{\sum }}\left\{\left({\tilde{\eta }}_{i}^{R}-\lambda_{1i}^{R}\right)|e_{1i}^{R}\left(t\right)||e_{1i}^{R}\left(t-\tau \left(t\right)\right)|\right\}+\overset{n}{\underset{i=1}{\sum }}\left\{\left({\tilde{\eta }}_{i}^{I}-\lambda_{1i}^{I}\right)|e_{1i}^{R}\left(t\right)||e_{1i}^{I}\left(t-\tau \left(t\right)\right)|\right\}\\ \;\leq -\overset{n}{\underset{i=1}{\sum }}\frac{G_{c}}{T_{c}}\left[\lambda_{2i}|e_{1i}^{R}\left(t\right){|}^{2}+\lambda_{3i}|e_{1i}^{R}\left(t\right){|}^{\alpha +1}+\lambda_{4i}|e_{1i}^{R}\left(t\right){|}^{\beta +1}\right],\\ \;\leq -\frac{G_{c}}{T_{c}}\left[\underset{i}{min}\left(\lambda_{2i}\right)\left(\overset{m}{\underset{i=1}{\sum }}|e_{1i}^{R}\left(t\right){|}^{2}\right)+\underset{i}{min}\left(\lambda_{3i}\right){\left(\overset{m}{\underset{i=1}{\sum }}|e_{1i}^{R}\left(t\right){|}^{2}\right)}^{\frac{\alpha +1}{2}}+n^{\frac{1-\beta }{2}}\cdot \underset{i}{min}\left(\lambda_{4i}\right){\left(\overset{m}{\underset{i=1}{\sum }}|e_{1i}^{R}\left(t\right){|}^{2}\right)}^{\frac{\beta +1}{2}}\right],\\ \;\leq -\frac{G_{c}}{T_{c}}\left[2\underset{i}{min}\left(\lambda_{2i}\right)\cdot V_{1}^{R}\left(t\right)+2^{\frac{\alpha +1}{2}}\underset{i}{min}\left(\lambda_{3i}\right)\cdot {\left(V_{1}^{R}\left(t\right)\right)}^{\frac{\alpha +1}{2}}+n^{\frac{1-\beta }{2}}\cdot 2^{\frac{\beta +1}{2}}\underset{i}{min}\left(\lambda_{4i}\right)\cdot {\left(V_{1}^{R}\left(t\right)\right)}^{\frac{\beta +1}{2}}\right]. \end{array} \end{array}$$

The proofs of $V_{1}^{I}\left(t\right)$, $V_{2}^{R}\left(t\right)$ and $V_{2}^{I}\left(t\right)$ are similar with that of $V_{1}^{R}\left(t\right)$. Additionally, we have


$$\begin{array}{l} {\dot{V}}_{1}^{I}(t)\leq -\frac{G_{c}}{T_{c}}[2\underset{i}{min}(\lambda_{2i})\cdot V_{1}^{I}(t)+2^{\frac{\alpha +1}{2}}\underset{i}{min}(\lambda_{3i})\cdot {\left(V_{1}^{I}(t)\right)}^{\frac{\alpha +1}{2}}\\ \;+n^{\frac{1-\beta }{2}}\cdot 2^{\frac{\beta +1}{2}}\underset{i}{min}(\lambda_{4i})\cdot {\left(V_{1}^{I}(t)\right)}^{\frac{\beta +1}{2}}],\\ {\dot{V}}_{2}^{R}(t)\leq -\frac{G_{c}}{T_{c}}[2\underset{i}{min}(k_{2j})\cdot V_{2}^{R}(t)+2^{\frac{\alpha +1}{2}}\underset{i}{min}(k_{3j})\cdot {\left(V_{2}^{R}(t)\right)}^{\frac{\alpha +1}{2}}\\ \;+m^{\frac{1-\beta }{2}}\cdot 2^{\frac{\beta +1}{2}}\underset{i}{min}(k_{4j})\cdot {\left(V_{2}^{R}(t)\right)}^{\frac{\beta +1}{2}}],\\ {\dot{V}}_{2}^{I}(t)\leq -\frac{G_{c}}{T_{c}}[2\underset{i}{min}(k_{2j})\cdot V_{2}^{I}(t)+2^{\frac{\alpha +1}{2}}\underset{i}{min}(k_{3j})\cdot {\left(V_{2}^{I}(t)\right)}^{\frac{\alpha +1}{2}}\\ \;+m^{\frac{1-\beta }{2}}\text{​}\cdot \text{​}2^{\frac{\beta +1}{2}}\underset{j}{min}(k_{4j})\cdot {\left(V_{2}^{I}(t)\right)}^{\frac{\beta +1}{2}}]. \end{array}$$

According to the analysis above, we can obtain that


$$\begin{array}{l} {\dot{V}}^{u}(t)\leq -\frac{2G_{c}}{T_{c}}\{\underset{i}{min}(\lambda_{2i})(V_{1}^{R}(t))+\underset{i}{min}(\lambda_{2i})(V_{1}^{I}(t))\\ \;+\underset{j}{min}(k_{2j})\cdot (V_{2}^{R}(t))+\underset{j}{min}(k_{2j})(V_{2}^{I}(t))\}\\ \;-\frac{2^{\frac{\alpha +1}{2}}G_{c}}{T_{c}}\{\underset{i}{min}(\lambda_{3i}){\left(V_{1}^{R}(t)\right)}^{\frac{\alpha +1}{2}}+\underset{i}{min}(\lambda_{3i}){\left(V_{1}^{I}(t)\right)}^{\frac{\alpha +1}{2}}\\ \;+\underset{j}{min}(k_{3j}){\left(V_{2}^{I}(t)\right)}^{\frac{\alpha +1}{2}}+\underset{j}{min}(k_{3j}){\left(V_{2}^{R}(t)\right)}^{\frac{\alpha +1}{2}}\}\\ \;-\frac{2^{\frac{\beta +1}{2}}G_{c}}{T_{c}}\{n^{\frac{1-\beta }{2}}\cdot \underset{i}{min}(\lambda_{4i}){\left(V_{1}^{R}(t)\right)}^{\frac{\beta +1}{2}}+n^{\frac{1-\beta }{2}}\cdot \underset{i}{min}(\lambda_{4i}){\left(V_{1}^{I}(t)\right)}^{\frac{\beta +1}{2}}\\ \;+m^{\frac{1-\beta }{2}}\cdot \underset{j}{min}(k_{4j}){\left(V_{2}^{R}(t)\right)}^{\frac{\beta +1}{2}}+m^{\frac{1-\beta }{2}}\cdot \underset{j}{min}(k_{4j}){\left(V_{2}^{I}(t)\right)}^{\frac{\beta +1}{2}}\}, \end{array}$$

Therefore,


$$\begin{array}{l} {\overset{∙}{V}}^{u}\left(t\right)\leq -\frac{2G_{c}}{T_{c}}\left\{\underset{i}{min}\left(\lambda_{2i}\right)\left[V_{1}^{R}\left(t\right)+V_{1}^{I}\left(t\right)\right]+\underset{j}{min}\left(k_{2j}\right)\cdot \left[V_{1}^{R}\left(t\right)+V_{1}^{I}\left(t\right)\right]\right\}\\ \;-\frac{2^{\frac{\alpha +1}{2}}G_{c}}{T_{c}}\left\{\underset{i}{min}\left(\lambda_{3i}\right){\left[V_{1}^{R}\left(t\right)+V_{1}^{I}\left(t\right)\right]}^{\frac{\alpha +1}{2}}+\underset{j}{min}\left(k_{3j}\right){\left[V_{2}^{R}\left(t\right)+V_{2}^{I}\left(t\right)\right]}^{\frac{\alpha +1}{2}}\right\}\\ \;-\frac{2^{\frac{\beta +1}{2}}G_{c}}{T_{c}}\left\{\underset{i}{min}\left(\lambda_{4i}\right)\cdot n^{\frac{1-\beta }{2}}\cdot {\left[V_{1}^{R}\left(t\right)+V_{1}^{I}\left(t\right)\right]}^{\frac{\beta +1}{2}}+\underset{i}{min}\left(k_{4j}\right)\cdot m^{\frac{1-\beta }{2}}\cdot {\left[V_{2}^{R}\left(t\right)+V_{2}^{I}\left(t\right)\right]}^{\frac{\beta +1}{2}}\right\},\\ \;\leq -\frac{2^{\frac{\alpha +1}{2}}\lambda \cdot G_{c}}{T_{c}}\cdot \left[{\left(V_{1}^{u}\left(t\right)\right)}^{\frac{\alpha +1}{2}}+{\left(V_{2}^{u}\left(t\right)\right)}^{\frac{\alpha +1}{2}}\right]-\frac{2\omega \cdot G_{c}}{T_{c}}\cdot \left[V_{1}^{u}\left(t\right)+V_{2}^{u}\left(t\right)\right]\\ \;-\frac{2^{\frac{\beta +1}{2}}\mu \cdot G_{c}}{T_{c}}\cdot \left[{\left(V_{1}^{u}\left(t\right)\right)}^{\frac{\beta +1}{2}}+{\left(V_{2}^{u}\left(t\right)\right)}^{\frac{\beta +1}{2}}\right],\\ \;\leq -\frac{G_{c}}{T_{c}}\left[2^{\frac{\alpha +1}{2}}\lambda \cdot {\left(V^{u}\left(t\right)\right)}^{\frac{\alpha +1}{2}}+2\mu \cdot {\left(V^{u}\left(t\right)\right)}^{\frac{\beta +1}{2}}+2\omega \cdot \left(V^{u}\left(t\right)\right)\right], \end{array}$$

where $a=\lambda \cdot 2^{\frac{\alpha +1}{2}}$, $\rho =\frac{\alpha +1}{2}$, *b* = 2μ, $\zeta =\frac{1+\beta }{2}$, *c* = 2ω, $\lambda =min\left\{\underset{i}{min}\left(\lambda_{3i}\right),\underset{j}{min}\left(k_{3j}\right)\right\}$, $\mu =min\left\{\underset{i}{min}\left(\lambda_{4i}\right)\cdot n^{\frac{1-\beta }{2}},\underset{j}{min}\left(k_{4j}\right)\cdot m^{\frac{1-\beta }{2}}\right\}$, $\omega =min\left\{\underset{i}{min}\left(\lambda_{2i}\right),\underset{j}{min}\left(k_{2j}\right)\right\}$.Under Theorem 2, the drive system (1) and the response system (2) can achieve predefined-time synchronization under the controller (15). The proof is completed.

** Remark 4**. In Theorems 2 and 3, the error system (5) can achieve predefined-time stability, in which *G*_*c*_ can be considered as the minimum upper bound *T*_*max*_ of the stability time in fixed-time stability. In addition, the theorem also provides a tuning parameter *T*_*c*_ to adjust the stability time to the expected value.

## 4. Numerical examples

Three examples are shown in this section. Example 1 demonstrates the effects of Theorem 1, Example 2 verifies the validity of the predefined-time synchronization in Theorems 2 and 3, and Example 3 is an application of image encryption and decryption.

** Example 1**. The simulation model is a two-dimensional MCVBAMNNs with time-varying delays, and it is shown as follows:


(16) $$\begin{array}{l} \{\begin{array}{l} {\dot{x}}_{1i}^{u}\left(t\right)=-\eta_{i}^{u}\left(x_{1i}^{u}\left(t-\tau \left(t\right)\right)\right)x_{1i}^{u}\left(t-\tau \left(t\right)\right)+\overset{2}{\underset{j=1}{\sum }}a_{ji}^{u}\left(x_{1i}^{u}\left(t\right)\right)f_{j}^{u}\left(x_{2j}^{u}\left(t\right)\right)\\ \;+\overset{2}{\underset{j=1}{\sum }}b_{ji}^{u}\left(x_{1i}^{u}\left(t-\tau \left(t\right)\right)\right)f_{j}^{u}\left(x_{2j}^{u}\left(t-\sigma \left(t\right)\right)\right),\\ {\dot{x}}_{2j}^{u}\left(t\right)=-\xi_{j}^{u}\left(x_{2j}^{u}\left(t-\sigma \left(t\right)\right)\right)x_{2j}^{u}\left(t-\sigma \left(t\right)\right)+\overset{2}{\underset{i=1}{\sum }}c_{ij}^{u}\left(x_{2j}^{u}\left(t\right)\right)g_{i}^{u}\left(x_{1i}^{u}\left(t\right)\right)\\ \;+\overset{2}{\underset{i=1}{\sum }}d_{ij}^{u}\left(x_{2j}^{u}\left(t-\sigma \left(t\right)\right)\right)g_{i}^{u}\left(x_{1i}^{u}\left(t-\tau \left(t\right)\right)\right). \end{array} \end{array}$$

The response system is


(17) $$\begin{array}{l} \{\begin{array}{c} {ẏ}_{1i}^{u}\left(t\right)=-\eta_{i}^{u}\left(y_{1i}^{u}\left(t-\tau \left(t\right)\right)\right)y_{1i}^{u}\left(t-\tau \left(t\right)\right)+\overset{2}{\underset{j=1}{\sum }}a_{ji}^{u}\left(y_{1i}^{u}\left(t\right)\right)f_{j}^{u}\left(y_{2j}^{u}\left(t\right)\right)\\ +\overset{2}{\underset{j=1}{\sum }}b_{ji}^{u}\left(y_{1i}^{u}\left(t-\tau \left(t\right)\right)\right)f_{j}^{u}\left(y_{2j}^{u}\left(t-\sigma \left(t\right)\right)\right)+u_{i}^{u}\left(t\right),\\ {ẏ}_{2j}^{u}\left(t\right)=-\xi_{j}^{u}\left(y_{2j}^{u}\left(t-\sigma \left(t\right)\right)\right)y_{2j}^{u}\left(t-\sigma \left(t\right)\right)+\overset{2}{\underset{i=1}{\sum }}c_{ij}^{u}\left(y_{2j}^{u}\left(t\right)\right)g_{i}^{u}\left(y_{1i}^{u}\left(t\right)\right)\\ +\overset{2}{\underset{i=1}{\sum }}d_{ij}^{u}\left(y_{2j}^{u}\left(t-\sigma \left(t\right)\right)\right)g_{i}^{u}\left(y_{1i}^{u}\left(t-\tau \left(t\right)\right)\right)+v_{j}^{u}\left(t\right), \end{array} \end{array}$$

where *i* = 1, 2, *j* = 1, 2; $T_{i}=T_{j}^{{\;}^{\prime }}=\varpi_{j}=\varpi_{j}^{{\;}^{\prime }}=0$, ${ℵ}_{i}={ℵ}_{i}^{{\;}^{\prime }}=1$; $\eta_{i}^{u}=\xi_{j}^{u}=1+i$; $f_{j}^{u}\left(z\right)=sin\left(\left|z\right|\right)$, $g_{i}^{u}\left(z\right)=cos\left(|z|-1\right)$; τ(*t*) = *t*+0.1*sin*(*t*), σ(*t*) = *t*−0.1*cos*(*t*). The initial values of system (16) are φ1^*R*^(*s*) = (1, 2.1)^*T*^, φ1^*I*^(*s*) = (1.3, −1)^*T*^, φ2^*R*^(*s*) = (0.4, 1.2)^*T*^, φ2^*I*^(*s*) = (1, 0.25)^*T*^. The initial values of system (17) are ϕ1^*R*^(*s*) = (0.9, −1)^*T*^, ϕ1^*I*^(*s*) = (1.1, 0.75)^*T*^, ϕ2^*R*^(*s*) = (0.5, −0.8)^*T*^, ϕ2^*I*^(*s*) = (−0.6, 1.4)^*T*^. The memristor-based connection weights are listed as follows:


$$\begin{array}{l} {Â}^{R}=\left(\begin{array}{cc} 0.8 & -0.3\\ 0.5 & 0.1 \end{array}\right),\;{Ǎ}^{R}=\left(\begin{array}{cc} -0.5 & 0.2\\ -0.6 & -1 \end{array}\right),\;{Â}^{I}=\left(\begin{array}{cc} -0.8 & 0.5\\ -0.8 & -1.2 \end{array}\right),\; \end{array}$$

$$\begin{array}{l} {Ǎ}^{I}=\left(\begin{array}{cc} 0.8 & 0.1\\ -0.1 & -1.1 \end{array}\right),\;{\hat{B}}^{R}=\left(\begin{array}{cc} 0.3 & 0.2\\ 0.7 & -0.6 \end{array}\right),\;{\overset{̬}{B}}^{R}=\left(\begin{array}{cc} 0.4 & 0.2\\ 0.3 & -0.4 \end{array}\right),\; \end{array}$$

$$\begin{array}{l} {\hat{B}}^{I}=\left(\begin{array}{cc} -0.9 & 0.7\\ -0.2 & 0.7 \end{array}\right),\;{\overset{̬}{B}}^{I}=\left(\begin{array}{cc} -1.2 & 0.8\\ -0.4 & 0.9 \end{array}\right),\;{Ĉ}^{R}=\left(\begin{array}{cc} -1 & 0.5\\ 0.8 & -1.3 \end{array}\right),\; \end{array}$$

$$\begin{array}{l} {Č}^{R}=\left(\begin{array}{cc} -1.8 & 0.8\\ 1.2 & -1.5 \end{array}\right),\;{Ĉ}^{I}=\left(\begin{array}{cc} -1.1 & 0.2\\ 1.1 & -1.3 \end{array}\right),\;{Č}^{I}=\left(\begin{array}{cc} -1.3 & 0.5\\ 1.0 & -1.2 \end{array}\right),\; \end{array}$$

$$\begin{array}{l} {\hat{D}}^{R}=\left(\begin{array}{cc} -1.5 & 0.4\\ 0.3 & -2 \end{array}\right),\;{Ď}^{R}=\left(\begin{array}{cc} -1.8 & 0.5\\ 0.1 & -1.5 \end{array}\right),\;{\hat{D}}^{I}=\left(\begin{array}{cc} -1.0 & 0.3\\ 0.2 & -1.5 \end{array}\right),\; \end{array}$$

$$\begin{array}{l} {Ď}^{I}=\left(\begin{array}{cc} -1.2 & 0.6\\ 0.5 & -1.8 \end{array}\right). \end{array}$$

**Figure 4:**
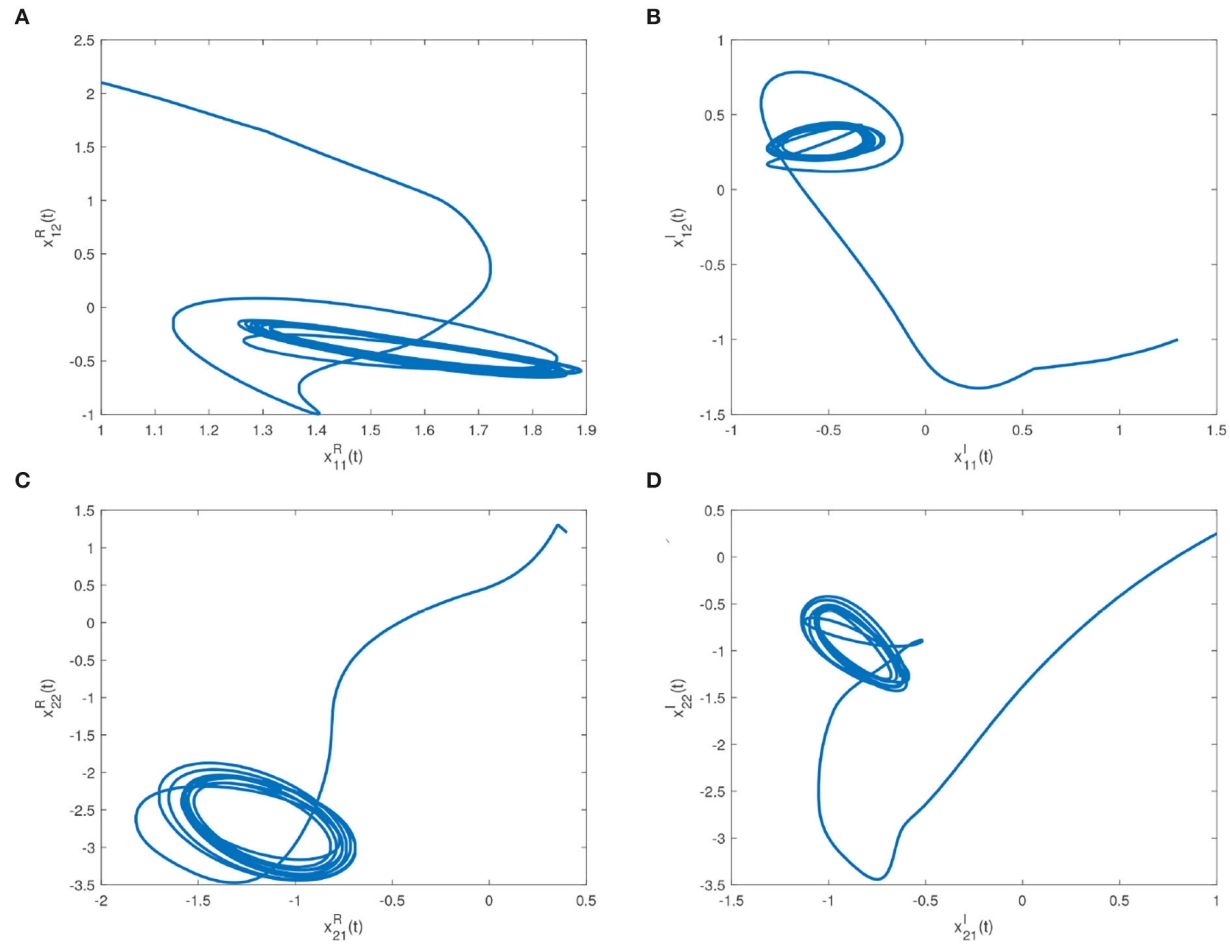
Real part and imaginary part phase plots of the drive system (16). **(A)** Phase plot of real part with initial conditions $x_{11}^{R}\left(0\right)=1$, $x_{12}^{R}\left(0\right)=2.1$. **(B)** Phase plot of imaginary part with initial conditions $x_{11}^{I}\left(0\right)=1.3$, $x_{12}^{I}\left(0\right)=-1$. **(C)** Phase plot of real part with initial conditions $x_{21}^{R}\left(0\right)=0.4$, $x_{22}^{R}\left(0\right)=1.2$. **(D)** Phase plot of imaginary part with initial conditions $x_{21}^{I}\left(0\right)=1$, $x_{22}^{I}\left(0\right)=0.25$.

**Figure 5:**
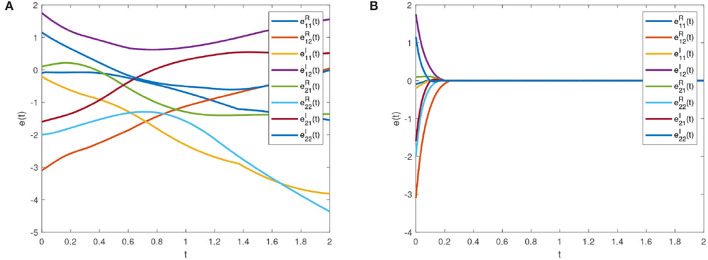
Error system trajectories of the drive system (16) and response system (17). **(A)** Synchronization errors without control. **(B)** Synchronization errors under control.

Some real and imaginary parts phase plots of the drive system (16) are shown in Figure 4. We choose *w*_*i*_ = 1.5, *k*_*i*_ = 1, *p*_*j*_ = 1.3, *q*_*j*_ = 1; $\lambda_{1i}^{R}=\lambda_{1i}^{I}=w_{1i}^{R}=w_{1i}^{I}=k_{1j}^{R}=k_{1j}^{I}=p_{1j}^{R}=p_{1j}^{I}=1$; λ_2*i*_ = 12, *k*_2*j*_ = 20; λ_3*i*_ = λ_4*i*_ = 0.4; *k*_3*j*_ = *k*_4*j*_ = 0.6. Errors of the drive system (16) and the response system (17) without and with feedback controller (14) are shown in Figures 5A,B, respectively.

Table 1 shows that $T_{max}^{1}$ is much smaller than $T_{max}^{2-4}$ with the same controller parameters. In Table 1, $T_{max}^{1}$ is derived by Theorem 1 and Lemma 3, $T_{max}^{2}$ is derived by Corollary 1 and Lemma 4, $T_{max}^{3}$ is derived by Corollary 2 and Lemma 5, and $T_{max}^{4}$ is derived by Corollary 3 and Lemma 6. Therefore, compared with Corollaries 1-3, Theorem 1 provides a more strict upper bound estimation formula.

**Table 1:** The comparisons among $T_{max}^{1}$, $T_{max}^{2}$, $T_{max}^{3}$, and $T_{max}^{4}$.

** $T_{max}^{1}$ **	** $T_{max}^{2}$ **	** $T_{max}^{3}$ **	** $T_{max}^{4}$ **
1.2	11.94	11.94	9.37

** Example 2**. According to Theorems 2-3, the settling time of the error system (5) can be adjusted by a tuning parameter *T*_*c*_.

We set two initial values:

(1) Initial value 1: φ1^*R*^(*s*) = (1, 2.1)^*T*^, φ1^*I*^(*s*) = (1.3, −1)^*T*^, φ2^*R*^(*s*) = (0.4, 1.2)^*T*^, φ2^*I*^(*s*) = (1, 0.25)^*T*^. ϕ1^*R*^(*s*) = (0.9, −1)^*T*^, ϕ1^*I*^(*s*) = (1.1, 0.75)^*T*^, ϕ2^*R*^(*s*) = (0.5, −0.8)^*T*^, ϕ2^*I*^(*s*) = (−0.6, 1.4)^*T*^;

(2) Initial value 2: φ1^*R*^(*s*) = (0.5, 2.5)^*T*^, φ1^*I*^(*s*) = (1.3, −1.5)^*T*^, φ2^*R*^(*s*) = (2, 1.25)^*T*^, φ2^*I*^(*s*) = (1.5, 0.25)^*T*^. ϕ1^*R*^(*s*) = (0.9, −1.5)^*T*^, ϕ1^*I*^(*s*) = (1.1, −1.2)^*T*^, ϕ2^*R*^(*s*) = (0.5, −1.8)^*T*^, ϕ2^*I*^(*s*) = (−0.6, 1.4)^*T*^.

**Figure 6:**
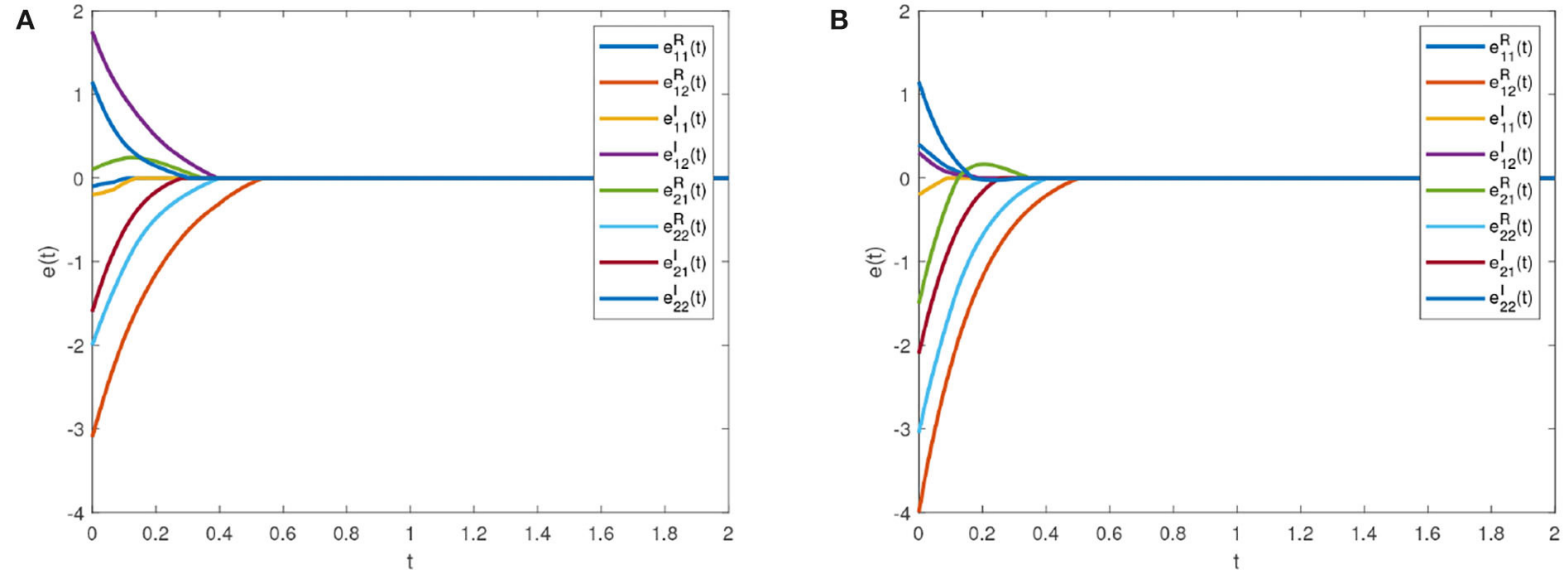
Synchronization errors with *T*_*c*_ = 5. **(A)** Synchronization errors with initial value 1. **(B)** Synchronization errors with initial value 2.

**Figure 7:**
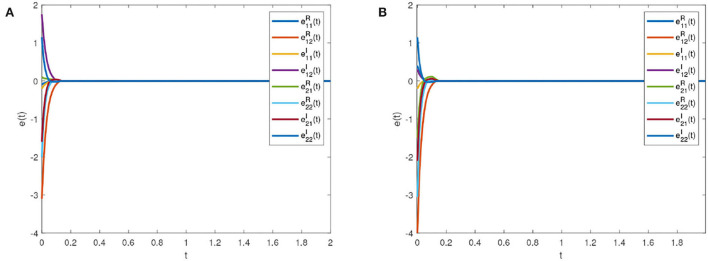
Synchronization errors with *T*_*c*_ = 0.5. **(A)** Synchronization errors with initial value 1. **(B)** Synchronization errors with initial value 2.

Figure 6 is the synchronization error diagram when *T*_*c*_ = 5. Figures 6A,B show that different initial values can achieve synchronization within a given time. Figure 7 is the synchronization error diagram when *T*_*c*_ = 0.5. From Figures 6, 7, it can be seen that the actual synchronization time is changed according to *T*_*c*_. We can set the ideal synchronization time *T*_*c*_ in the controller, which is that the system can achieve predefined-time stability.

** Example 3**. We use the predefined-time stability and chaotic characters of the drive system (16) and response system (17) to achieve image encryption and decryption. The proposed image encryption algorithm consists of pixel scrambling and diffusion.

We choose *T*_*c*_ = 0.2, and the other parameters are the same as in Example 1. According to the drive system (16), our encryption algorithm is designed as follows.

** Step 1**. Enter the color original image “Lena” with the size of *M* × *N* × 3, where *M* = 256, *N* = 256.

** Step 2**. According to $x_{11}^{u}\left(t\right)$ of the drive system (16), we can get the real part sequence and imaginary part sequence $x_{11\left(k1\right)}^{R}=\left[x_{11\left(1\right)}^{R},x_{11\left(2\right)}^{R},...,x_{11\left(M\times N/2\right)}^{R}\right]$, $x_{11\left(k2\right)}^{I}=\left[x_{11\left(1\right)}^{I},x_{11\left(2\right)}^{I},...,x_{11\left(M\times N/2\right)}^{I}\right]$. Based on the descending order of chaotic sequences $x_{11\left(k1\right)}^{R}$ and $x_{11\left(k2\right)}^{I}$, the index of the sequences ϱ_1_ and ϱ_2_ is obtained.


$$\begin{array}{l} \varrho_{1}=sort(round(x_{11(k1)}^{R},-3){,}^{\prime }descens^{\prime }),\\ \varrho_{2}=sort(round(x_{11(k2)}^{I},-3){,}^{\prime }descens^{\prime }). \end{array}$$

ϱ_1_ is used to scramble half of the original image, and ϱ_2_ is used to scramble the other half of the original image.

** Step 3**. Scramble the pixels of *R, G, B* channels and compose new $\tilde{R},\tilde{G},\tilde{B}$ channels.


$$\begin{array}{l} \tilde{R}=reshape(R,M,N)\tilde{G}=reshape(G,M,N)\\ \;\tilde{B}=reshape(B,M,N). \end{array}$$

** Step 4**. Converts $x_{12\left(k\right)}^{R}$ and $x_{12\left(k\right)}^{I}$ of the drive system (16) into *M*×*N* dimension matrices *z*1(*i, j*) and *z*2(*i, j*). Encrypt the $\tilde{R}$ channel as follows. For even-row pixels,


$$\begin{array}{l} z1(i,j)=mod(10^{8}(z1(k)-floor(z1(k))),256);\\ \;newR(i,j)=bitxor(\tilde{R}(i,j),floor(z1(i,j))); \end{array}$$

for odd-row pixels,


$$\begin{array}{l} z2(i,j)=mod(10^{8}(z1(k)-floor(z2(k))),256);\\ \;newR(i,j)=bitxor(\tilde{R}(i,j),floor(z2(i,j))); \end{array}$$

use *x*_21_ and *x*_22_ to encrypt $\tilde{G},\tilde{B}$ channels according to the above method, respectively.

**Figure 8:**
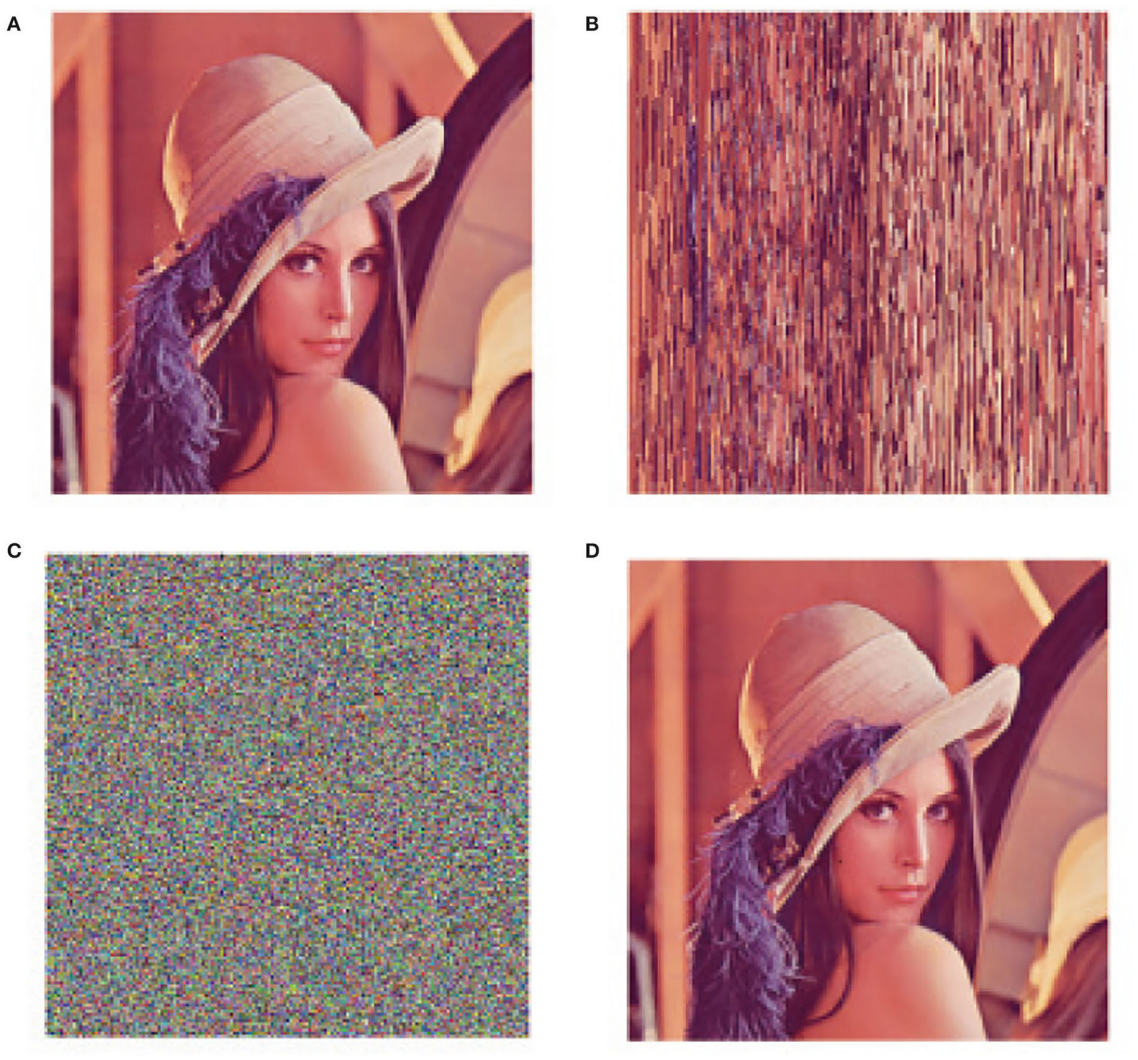
Original encryption and decryption pictures. **(A)** Original picture “Lena.” **(B)** Pixel scrambling picture. **(C)** The encrypted picture. **(D)** The decrypted picture (This image is taken from a public database).

The original picture is shown in Figure 8A. We use a chaotic sequence to scramble the pixels in the encrypted area, as shown in Figure 8B. The final encrypted image is shown in Figure 8C. When the drive system (16) and the response system (17) reach predefined-time synchronization, decryption is the opposite process of encryption, and the decrypted picture is shown in Figure 8D.

**Figure 9:**
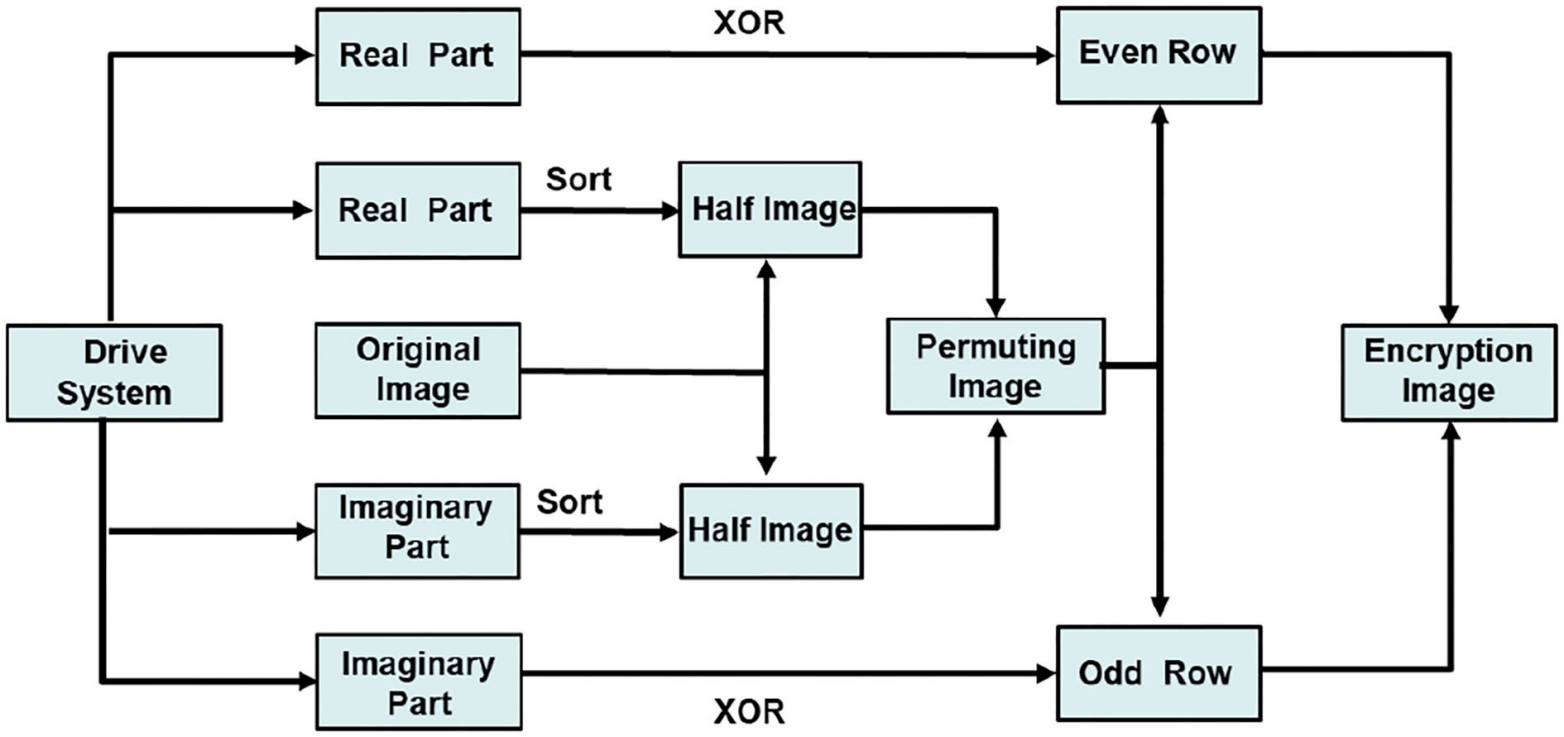
Flow chart of the encryption algorithm.

**Figure 10:**
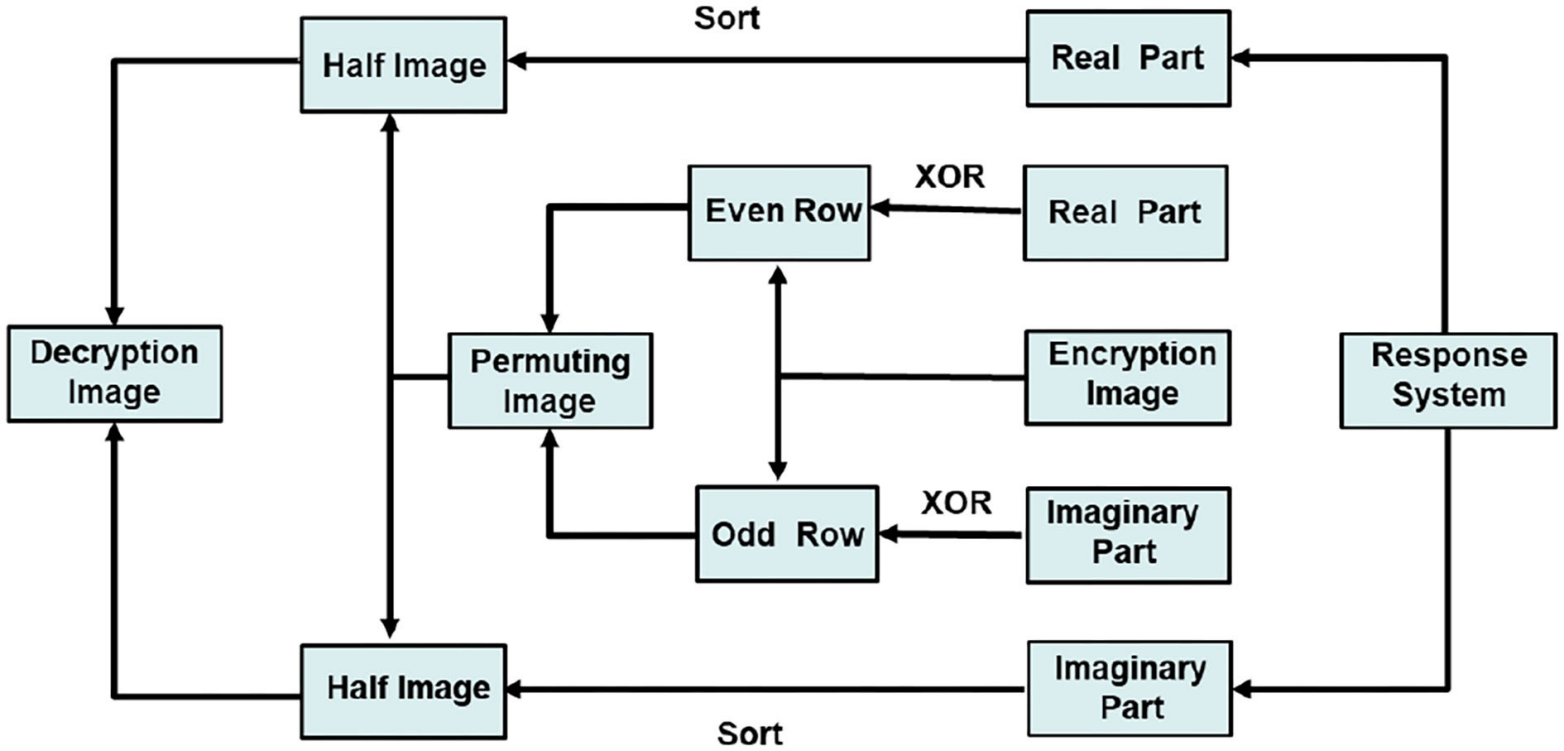
Flow chart of the decryption algorithm.

According to MCVBAMNNs and predefined-time synchronization, the flow charts of image encryption and decryption are shown in Figures 9, 10. We use the controller (15) to flexibly set the parameter *T*_*c*_, then the chaotic sequence can be selected in a controllable range, which ensures the effectiveness of the encryption and decryption algorithm. In this scheme, *T*_*c*_ = 0.2 is chosen as the secret key, and the wrong secret key will affect the decryption result.

**Figure 11:**
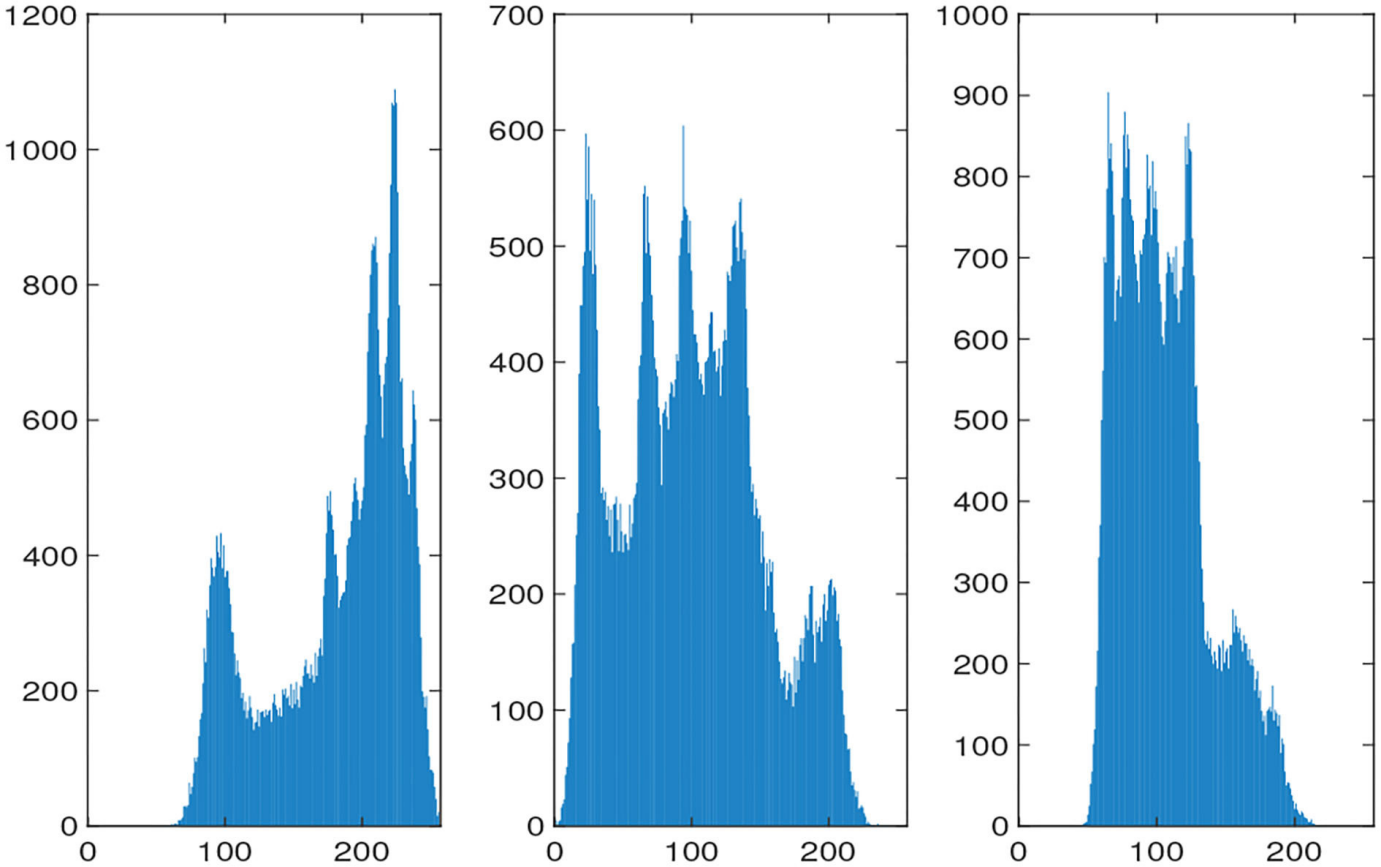
Histograms of RGB for the original picture.

**Figure 12:**
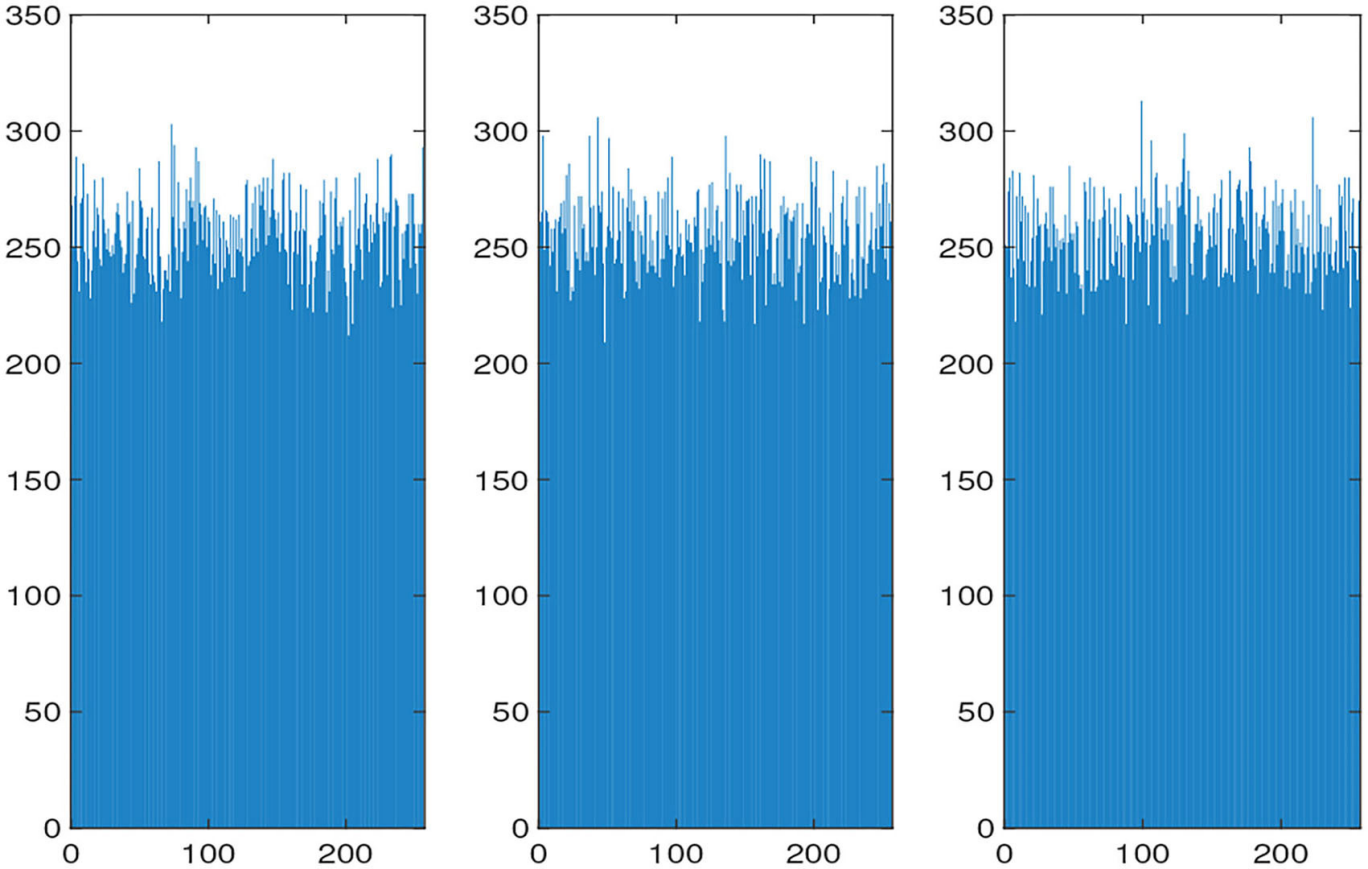
Histograms of RGB for the encrypted picture.

**Figure 13:**
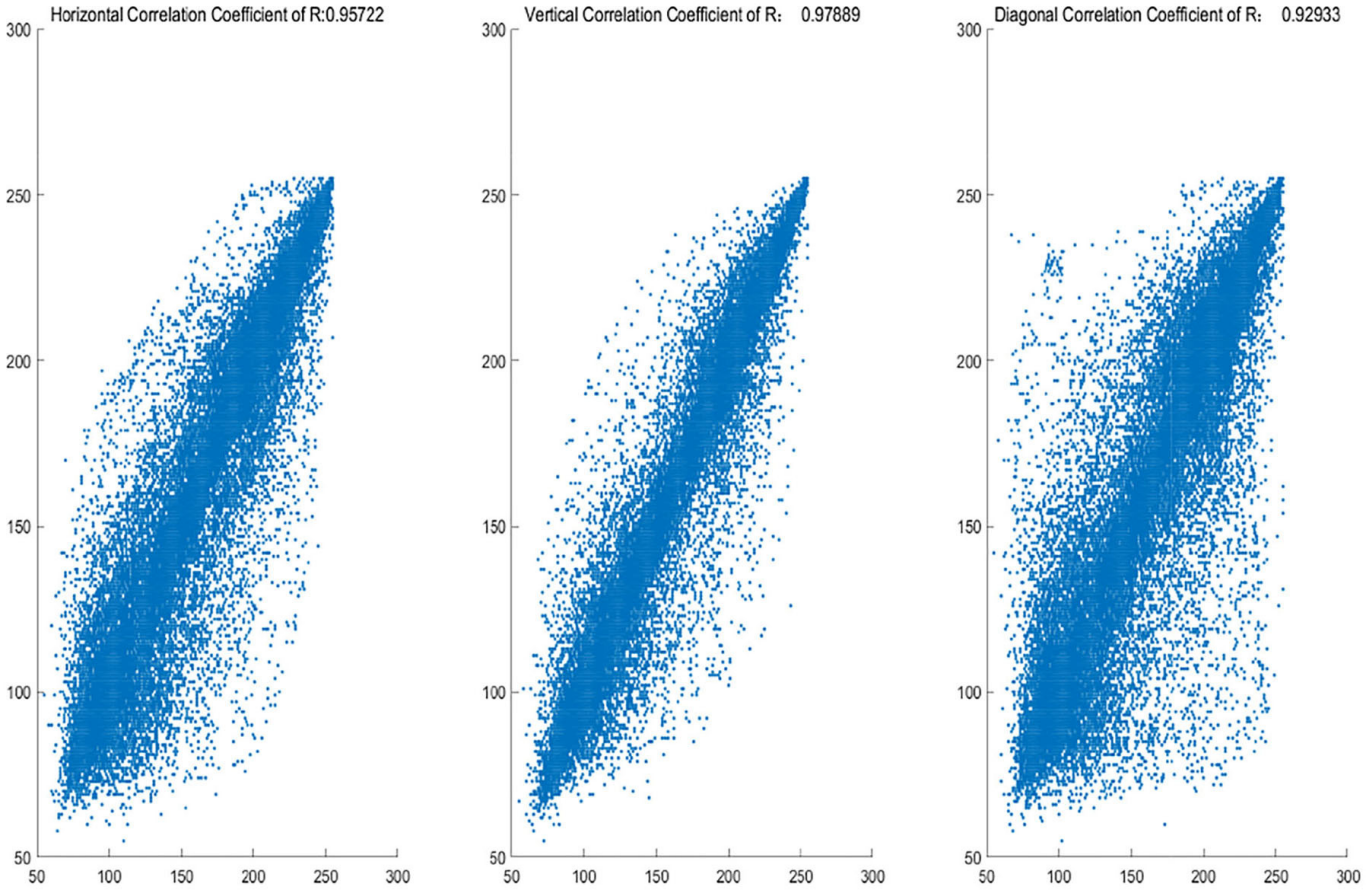
R-channel correlation coefficients of the original image (From left to right: Horizontal Correlation Coefficient of R: 0.95722; Vertical Correlation Coefficient of R: 0.9789; Diagonal Correlation Coefficient of R: 0.92933).

**Figure 14:**
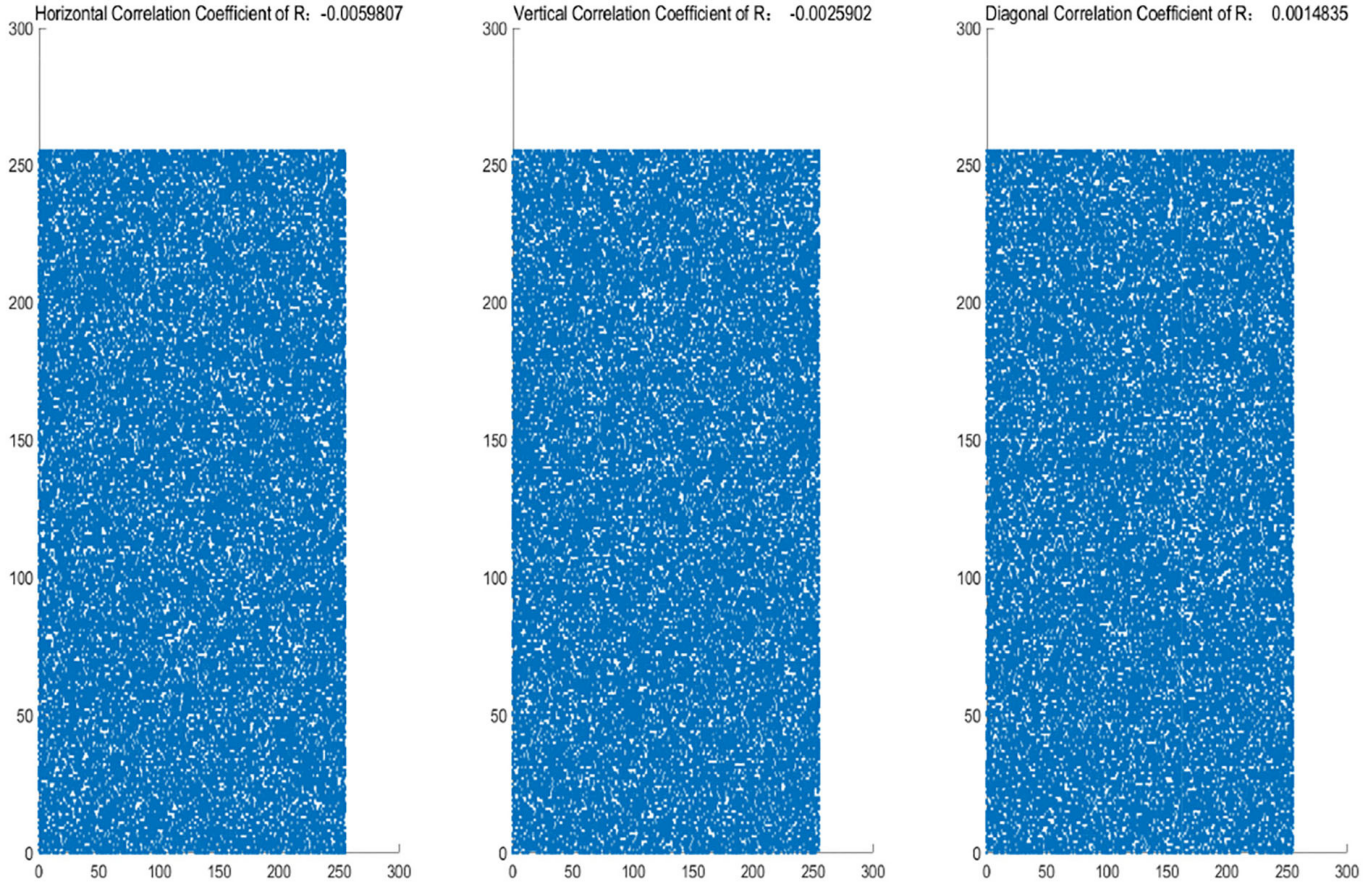
R-channel correlation coefficients of encryption image (from left to right:Horizontal Correlation Coefficient of R:-0.0059807; Vertical Correlation Coefficient of R: -0.0025902; Diagonal Correlation Coefficient of R: 0.0014835).

The histograms of the original and encrypted image are shown in Figures 11, 12, which shows that the histograms of the encryption image become highly disordered. In a digital image, there is a high correlation between each pixel. Therefore, the pixel adjacency correlation of encrypted images generated by a reasonable encryption algorithm should be close to zero. The horizontal correlation coefficients of the original and the encryption image in the R are shown in Figures 13, 14. The correlation coefficient of the encryption image becomes much lower. Table 2 lists the correlation coefficients of the encryption algorithm and the comparisons with others, which indicates our algorithm has good results^1^.

**Table 2:** Comparison of correlation coefficients of encryption “Lena.”

	**H**	**V**	**D**
Original image	0.94295	0.96873	0.91310
Our algorithm	–0.0057	–0.0008	–0.0009
Liu et al. (2019)	–0.0087	–0.02116	–0.00381
Xu et al. (2014)	0.01190	0.01806	0.0678
Wu et al. (2015)	–0.0084	0.0004	–0.0015
Chen et al. (2016)	–0.0043	–0.0037	0.0196

**Figure 15:**
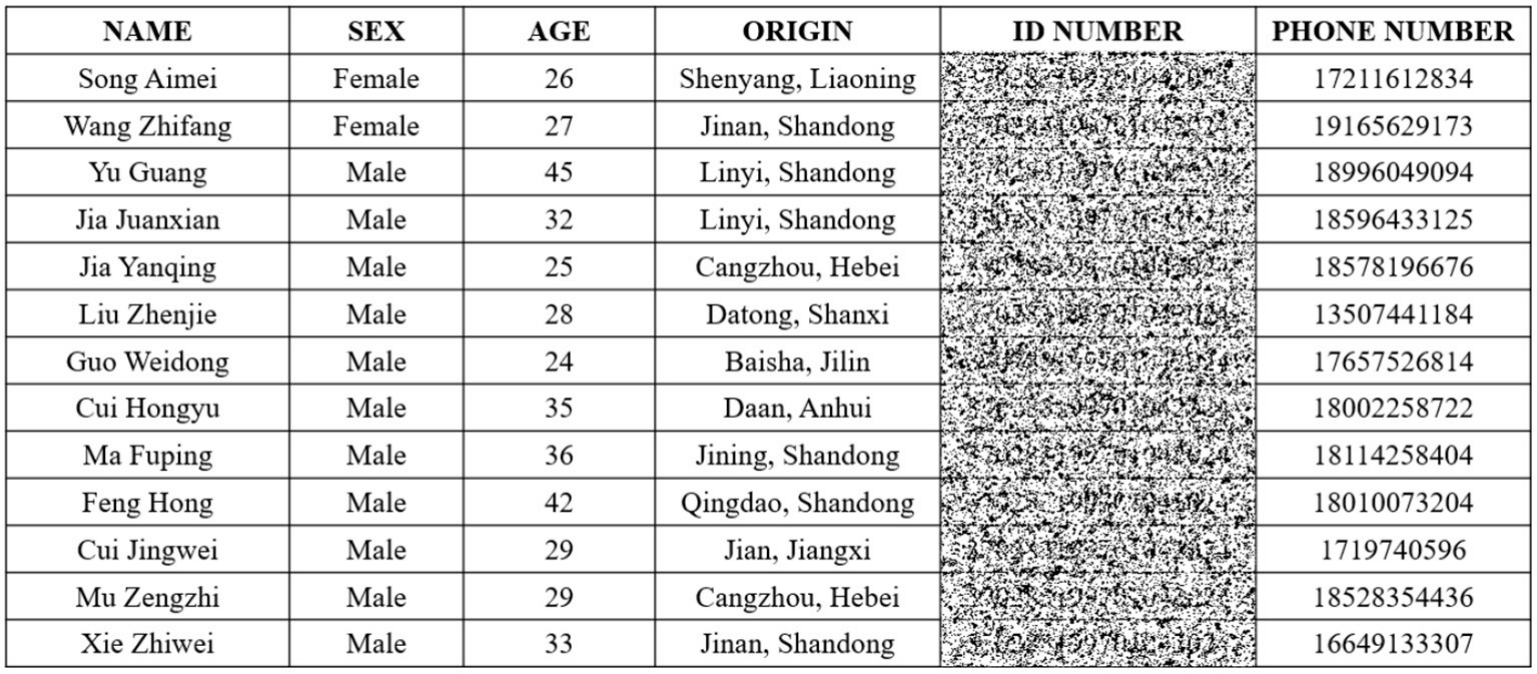
Employee information diagram of a company. (Encrypted ID Number).

** Remark 5**. Digital images can convey information intuitively and effectively and are widely used. A considerable part of images in daily life and work contains sensitive data and belong to sensitive areas. If the sensitive block data is not protected, it may cause some losses to individuals or other objects. Therefore, the image encryption and decryption scheme based on the predefined-time synchronization of MCVBAMNNs in this paper has important application value for the privacy protection of image blocks with uncertain size without losing image availability. For example, Figure 15 is a picture containing employee information. In order to avoid privacy disclosure, important information such as ID numbers or mobile phone numbers can be encrypted.

** Remark 6**. At present, most of the neural network dynamics achievements are still in the theoretical stage, and the research on relevant practical applications is not extensive enough. But fortunately, researchers are aware of this problem and are trying to explore its future research direction, such as its application in human-computer interaction (Su et al., 2022).

## 5. Conclusion

In this paper, the fixed-time and predefined-time stability of MCVBAMNNs with leakage time-varying delay is studied. Based on differential inclusion and set-valued mapping theory, an effective discontinuous controller is designed, sufficient conditions for conservative smaller fixed-time synchronization are obtained, and a more general predefined-time stability theorem is proposed. By adjusting the controller parameters, the MCVBAMNNs can achieve synchronization within a predetermined time. On this basis, we design an effective image encryption scheme. Through comparative analysis, the algorithm proposed in this paper has good results. Inspired by Feng et al. (2020), in the future, we will consider the method of complex-valued nonseparation and propose more general predefined-time stability conditions, which will be an interesting and challenging job.

## Data availability statement

The original contributions presented in the study are included in the article/supplementary material, further inquiries can be directed to the corresponding author/s.

## Author contributions

AL: formal analysis, validation, and writing—original draft. HZ: data curation and funding acquisition. QW: data curation. SN: funding acquisition and supervision. XG, ZS, and LL: funding acquisition and supervision. All authors contributed to the article and approved the submitted version.

## Funding

This study is supported by the National Natural Science Foundation of China (Grant Nos. 62103165, 62032002, 62101213, and 61902048), the Natural Science Foundation of Shandong Province (Grant No. ZR2020QF107), the Natural Science Foundation of Beijing Municipality (Grant No. M21034), the Chongqing High Tech Research Program (Grants No. cstc2018jcyjAX0279), and Development Program Project of Youth Innovation Team of Institutions of Higher Learning in Shandong Province.

## Conflict of interest

The authors declare that the research was conducted in the absence of any commercial or financial relationships that could be construed as a potential conflict of interest.

## Publisher's note

All claims expressed in this article are solely those of the authors and do not necessarily represent those of their affiliated organizations, or those of the publisher, the editors and the reviewers. Any product that may be evaluated in this article, or claim that may be made by its manufacturer, is not guaranteed or endorsed by the publisher.

## Footnotes

^1^Code is available at: https://github.com/Liu-aidi/MCVBAMNNs.
